# Immediate-Early Promoter-Driven Transgenic Reporter System for Neuroethological Research in a Hemimetabolous Insect

**DOI:** 10.1523/ENEURO.0061-18.2018

**Published:** 2018-09-04

**Authors:** Takayuki Watanabe, Atsushi Ugajin, Hitoshi Aonuma

**Affiliations:** 1 Department of Biological Sciences, Faculty of Science, Hokkaido University, Kita 10 Jo Nishi 6 Chome, Kita-Ku, Sapporo 060-0810, Japan; 2 Laboratory of Evolution of Insect Feeding Habit, JT Biohistory Research Hall, Takatsuki 569-1125, Japan; 3 Research Center of Mathematics for Social Creativity, Research Institute for Electronic Science, Hokkaido University, Sapporo 060-0811, Japan; 4 CREST, Japan Science and Technology Agency, Kawaguchi 332-0012, Japan

**Keywords:** Activity mapping, *Gryllus bimaculatus*, immediate-early gene, transgenesis

## Abstract

Genes expressed in response to increased neuronal activity are widely used as activity markers in recent behavioral neuroscience. In the present study, we established transgenic reporter system for whole-brain activity mapping in the two-spotted cricket *Gryllus bimaculatus*, a hemimetabolous insect used in neuroethology and behavioral ecology. In the cricket brain, a homolog of *early growth response-1* (*Gryllus egr-B*) was rapidly induced as an immediate-early gene (IEG) in response to neuronal hyperexcitability. The upstream genomic fragment of *Gryllus egr-B* contains potential binding sites for transcription factors regulated by various intracellular signaling pathways, as well as core promoter elements conserved across insect/crustacean *egr-B* homologs. Using the upstream genomic fragment of *Gryllus egr-B,* we established an IEG promoter-driven transgenic reporter system in the cricket. In the brain of transgenic crickets, the reporter gene (a nuclear-targeted destabilized EYFP) was induced in response to neuronal hyperexcitability. Inducible expression of reporter protein was detected in almost all neurons after neuronal hyperexcitability. Using our novel reporter system, we successfully detected neuronal activation evoked by feeding in the cricket brain. Our IEG promoter-driven activity reporting system allows us to visualize behaviorally relevant neural circuits at cellular resolution in the cricket brain.

## Significance Statement

Insects are the largest and most diverse group of organisms and show a wide variety of behaviors. Despite the importance of a comparative approach, recent insect neuroethology mostly relies on the fruit fly *Drosophila melanogaster*, a versatile model insect with particularly powerful genetic tools, and a relatively small number of researchers use non-*Drosophila* insects. In the present study, a novel genetic reporting system for whole-brain activity mapping was established in the two-spotted cricket *Gryllus bimaculatus* by using the newly identified and evolutionary conserved gene regulatory region of an immediate-early gene, *early growth response*. This reporting system allowed us to analyze the behaviorally evoked neural activity patterns at cellular resolution in the cricket brain.

## Introduction

Insects are the largest and most diverse group of organisms on Earth. They originated ∼500 million years ago, at almost the same time as the first terrestrial plants (Misof et al., 2014). Now, they represent ∼80% of all living organisms and are found in almost all terrestrial and freshwater environments. As a result of their evolution and diversification, insects show a wide variety of behaviors, including locomotion, feeding, molting, diapause, and social behaviors (e.g., agonistic interaction, courtship and mating behavior). Therefore, insects have been widely used for neuroethological studies over the past decades.

Recent insect neuroethology largely relies on the fruit fly *Drosophila melanogaster*, a versatile model organism with particularly powerful neurogenetic tools [e.g., an abundant collection of transgenic lines (Jenett et al., 2012; Manning et al., 2012), recombination-based genetic systems for targeted gene expression in specific neural circuits (Lee and Luo, 2001; Lee, 2014; Griffin et al., 2014), optogenetic/thermogenetic tools and genetically encoded calcium indicators (Owald et al., 2015; Riemensperger et al., 2016)], which allow us to manipulate/monitor the activity of specific neural circuits during behavior. In contrast, a relatively small number of researchers use non-*Drosophila* insects despite the importance of a comparative approach to understanding the general principles and evolution of insect behavior and its underlying neural mechanisms. Without sophisticated genetic tools, researchers have to depend on limited experimental techniques such as electrophysiology, neuroimaging, and behavioral pharmacology. Although these classic techniques are well established, they have some inevitable weaknesses and limitations: for example, neurophysiological techniques are vulnerable to mechanical disturbances caused by animal movements, and pharmacology provides us limited information on the involvement of receptors/signaling cascades in a certain behavior. To facilitate neuroethological studies in non-*Drosophila* insects, a technical breakthrough is necessary to compensate/overcome these problems. In recent behavioral neuroscience, histologic detection of activity-regulated genes has been widely employed for retroactive labeling of behaviorally relevant neural circuits (Clayton, 2000; Guzowski et al., 2005; Maruska et al., 2013; Kawashima et al., 2014). In this study, aiming to introduce such a powerful technique to non-*Drosophila* insects, we established a novel transgenic reporter system for whole-brain activity mapping in a hemimetabolous insect.

In neurons, the expression of a wide variety of genes is regulated in response to increased neuronal excitation and activation of cellular signaling pathways (Flavell and Greenberg, 2008), which are involved in various cellular functions, including transcription regulation, signaling pathways, metabolism, and synaptic function (Loebrich and Nedivi, 2009; Okuno, 2011). In terms of expression time course and regulatory mechanisms, activity-regulated genes can be classified into two categories, immediate-early gene (IEGs) and delayed-early genes (DEGs). The neuronal IEGs are rapidly and transiently induced in response to neuronal activation and their expression does not require *de novo* protein synthesis (Clayton, 2000). Some IEGs encode transcription factors whose protein products, in turn, regulate expression of DEGs. Therefore, transcription factor IEGs are considered as the first wave of genomic response to shape the cellular response toward various physiological/environmental stimuli. In the nervous system, transcription factor IEGs, such as *c-fos* and *egr-1/zif268/NGFI-A*, are expressed in response to a wide variety of stimuli and in multiple cell types; therefore, they are widely used for activity mapping in the vertebrate system. In the early studies, researchers directly detected the activity-regulated expression of gene products of IEGs to map behaviorally evoked neuronal activity. In the recent studies, the promoter regions of activity-regulated genes were used in combination with optogenetic tools to address the functional importance of behaviorally relevant circuits (Ramirez et al., 2014; Minatohara et al., 2016).

So far, activity-regulated gene–based activity mapping has been conducted on limited insect species, such as the honeybee, *Drosophila*, and silk moth. In the brain of the honeybee, the noncoding RNA *kakusei* and a homolog of transcription factor c-*jun, jun-related antigen* (*jra*), were used to map a neural activity associated with the foraging behavior and waggle dance (Kiya et al., 2007; Kiya and Kubo, 2011) and defensive behaviors (Alaux and Robinson, 2007; Ugajin et al., 2012). In the brain of *Drosophila* and the silk moth, a nuclear receptor *hr38* (*NGFI-B/nur77/NR4A1* homolog) was used for activity mapping (Fujita et al., 2013). In contrast, there has been no report on the activity-regulated genes or neuronal IEGs in basal hemimetabolous insects. Moreover, the activity-regulated promoter has not been characterized in any insect species.

Crickets have been widely used for neuroethology and behavioral ecology for decades (Huber et al., 1989; Horch et al., 2017) with a particular focus on their prominent social behaviors including courtship and aggressive behaviors (Adamo et al., 1995; Pollack, 2000; Hedwig, 2006; Stevenson and Schildberger, 2013). Moreover, modern genetic techniques (e.g., transposon-mediated transgenesis, genome editing techniques) have been introduced to the two-spotted cricket *Gryllus bimaculatus* over the last decade (Nakamura et al., 2010; Watanabe et al., 2012; Horch et al., 2017). For this reason, we chose *G. bimaculatus* as a model system to establish a transgenic activity reporting system using an activity-regulated promoter. In the present study, we first identified neuronal IEGs in the cricket to isolate and characterize the activity-regulated promoter in basal insects. We then established a transgenic reporter line for a whole-brain, single cell–resolution activity mapping, which is required for further functional studies of behaviorally relevant neural circuits.

## Materials and Methods

### Animals

A wild-type strain of two-spotted crickets *G. bimaculatus* DeGeer was inbred for decades in our laboratory (Hokudai WT strain). The Hokudai WT strain was used for identification and initial expression analysis of the candidate IEGs, and promoter analysis. A congenic white-eye strain (Hokudai *gwhite* strain) was established by five-time backcrossing of *gwhite* mutant (kindly provided by Prof. Noji in Tokushima Univ., Japan) to the Hokudai WT strain. The Hokudai *gwhite* strain was used for transgenesis. Crickets were reared in a group on a 14-h:10-h light/dark cycle at 28°C. They were fed a diet of insect food pellet (Oriental Yeast Co.) and water *ad libitum*. Specimens of the European honeybee *Apis mellifera* L. were collected from colonies maintained at Tamagawa University. Specimens of the house cricket *Acheta domesticus* were purchased at a local pet store. Specimens of the katydid *Gampsocleis buergeri* and migratory locust *Locusta migratoria* were collected in Tokyo (Japan).

### Identification of candidate neuronal IEGs

We amplified cDNAs encoding a full-length protein coding sequence of three candidate IEGs and a partial cDNA of *Gryllus hr38* from cDNA libraries derived from the brains of adult crickets prepared in Watanabe et al. (2011).

#### Partial cDNA cloning of Gryllus fra

Partial cDNA of *Gryllus fra* was amplified using gene-specific primers (GSPs) designed on the basis of *G. bimaculatus* expressed sequence tag (EST) clones corresponding to *fra* (GenBank IDs AK282142 and AK254978).

#### Partial cDNA cloning of Gryllus jra

Partial cDNA of *Gryllus jra* was amplified using GSPs designed on the basis of a *G. bimaculatus* EST clone corresponding to *Gryllus jra* (GenBank ID AK263019).

#### Partial cDNA cloning of Gryllus egr

Partial cDNA of *Gryllus egr* was amplified using degenerate primers. Degenerate primers were designed on the basis of conserved amino acid sequences (GVQLAEY, TSKGHEI, FQCRICMR and HAKVHLK) among insect Egr homologs. Sequences of the degenerate primers are listed in Table 1.

**Table 1.** Nucleotide sequences of primers used in this study

	Forward primer	Reverse primer
**Degenerate primers**		
*egr*	5′-GGA GTN CAR CTN GCH GAR TA-3′	5′-GAN CGC ATG CAD ATN CGR CAY TG-3′
	5′-ACS AGN AAR GGN CAY GAR AT-3′	5′-TTN AGR TGN ACY TTN GCR TG-3′
	5′-CCA RTG YCG NAT HTG CAT GCG-3′	
*hr38*	5′-AAC CGC TGC CAR TTY TGC-3′	5′-AGA AGC TCC TGR TCR TNG C-3′
*tdc2*	5′-TCG AGT ACG CSG AYT CKT TCA ACA C-3′	5′-GGA TCR CTS ACC ATN CGN ACG AAG AA-3′
**Primers for full-length ORF amplification**		
*fra-A*	5′-CGC GGG AGT AAG GAC GTG-3′	5′-CCC CAT TGT CCA AAT CCT CC-3′
*fra-B*	5′-GGC GGC TTG TGT GTT TGT G-3′	5′-CCC CAT TGT CCA AAT CCT CC-3′
*jra*	5′-GAC GGT CGC GGA GAG TC-3′	5′-GAT CTC ATA TGT ATA TGC ATG TGT TCA C-3′
*egr-B*	5′-TTC ATT CAT AAA AGT GTT GTA GAG CG-3′	5′-ATA TAT ACG AAT CGA GGA GAA CAC-3′
*tdc1*	5′-CAT CTG GCG TTC GCT C-3′	5′-CGC AGT CCC AGA AGA G-3′
*tdc2*	5′-CGA CGC CCG ACG ACA TTC G-3′	5′-CCG GCT CGT ATG TTG TGT GG-3′
**Primers for RT-qPCR**		
*fra* total transcripts	5′-GGA CGG CCT CAA TTC GGG-3′	5′-GGA TTC CAC CTC GCA CTG C-3′
*fra*-*A*	5′-CCT GCC TTC ATC TGC GTA CG-3′	5′-GTC TCA CTG GGC GAA ACG TG-3′
*fra*-*B*	5′-GGC GGC TTG TGT GTT TGT G-3′	5′-GGA TTC CAC CTC GCA CTG C-3′
*jra*	5′-GAG CGG ACG GTT GTG TTA GG-3′	5′-GCA GTT GCG TAC CAT CTA AAT CC-3′
*egr-B* (for initial expression analysis)	5′-GAC CTA GGC GTC GAA CCC-3′	5′-GTT CCA AGG ATC CTG TGA TGG G-3′
*egr-B*	5′-GTT TGG AAA CGC TGA GCC C-3′	5′-CCT GAC GCT GTA GAG GCA C-3′
*egr-B* pre-mRNA	5′-GTG ACA CAT GTA ATT GGC GTA AC-3′	5′-CAA TTC CTC GGG TTC CAA GG-3′
*hr38*	5′-CCA ACC TCG ACT ATT CAC AGT ATC-3′	5′-CCG GAA TCT TAT CAG CAA ACG TG-3′
*hr38* pre-mRNA	5′-GAA GCA TCT ACT CCA GTC TCA TAA TAG-3′	5′-GTA GGC TCA CGA TAC TGG AAA TG-3′
*EYFPnls:PEST*	5′-CGA GGA GCT GTT CAC CGG-3′	5′-GGT GCA GAT GAA CTT CAG GG-3′
*β-actin*	5′-CGT AAA CTC AAC TAC TAA CCA TGT GC-3′	5′-GCC CTG GGT GCA TCA TCG-3′
*ef1α*	5′-CGA CTC CGG TAA ATC TAC GAC C-3′	5′-CAC CCA GGC ATA CTT GAA AGA AC-3′
*rpl32*	5′-CGC CCA GTT TAT CGT CCA AC-3′	5′-GCC TGC GAA CTC TGT TGT C-3′
**Primers used to amplify the core promoter regions of orthopteran *egr-B* homologs**		
	5′-GTT ACG TCA TTT GAC GTC A-3′	5′-GTC CCA TAT TTG GAA GTC G-3′
	5′-GTT ACG TCA TTT TGA CGT CA-3′	5′-CAM CAS TTT TAT GAA TGA AG-3′
**Primers used to amplify the genomic DNA fragment upstream to the coding sequence of the orthopteran *egr-B* homologs**		
*Gryllus bimaculatus*	5′-CAG GGG TTG TTT ATT CGC CG-3′	5′-CTG TGA TGG GAG GCG GTT CAA C-3′
*Acheta domesticus*	5′-AAA TTC GAA AGC CTT GAC AGT GG-3′	5′-ACG ATG GAC GAG CGT CGT G-3′
*Gampsocleis buergeri*	5′-ATG TTC CCC CTC CAT GCC AG-3′	5′-ACA TGC TGA CGC GCA ACA C-3′
*Locusta migratoria*	5′-CAG TGT TGC CAG CCT CC-3′	5′-CCG ACG AGT ACA GGC AGT C-3′

#### Partial cDNA cloning of Gryllus hr38

Partial cDNA of *Gryllus hr38* was amplified using degenerate primers. Degenerate primers were designed on the basis of conserved amino acid sequences (NRCQFC and RDDQELL) among insect *hr38* homologs. Sequences of the degenerate primers are listed in Table 1.

#### Full-length cDNA cloning of Gryllus fra, jra, and egr-B

First, nucleotide sequences flanking the translational initiation and termination sites of each gene were obtained by 5′ and 3′ rapid amplification of cDNA ends (RACEs) using the FirstChoice RLM-RACE kit (Ambion). RACE PCR was conducted using GSPs designed on the basis of the nucleotide sequences of partial cDNAs of each gene. Complementary DNAs containing full-length ORF of the genes were amplified using GSPs designed at the 5′ and 3′ untranslated region of the genes. Primers used to amplify cDNAs containing full-length ORF are listed in Table 1. All PCRs were performed using the Q5 High-Fidelity DNA polymerase (New England Biolabs). Amplified cDNA fragments were cloned into the pGEM-T Easy vector (Promega), and their nucleotide sequences were determined.

### Sequence comparison and structural analysis of the proteins

The deduced amino acid sequences of *Gryllus fra*, *jra, egr*, and *hr38* were aligned with those of the corresponding parts of homologous genes of other species using the MAFFT (Katoh and Standley, 2013; RRID: SCR_011811) or MUSCLE algorithms (Edgar, 2004; RRID: SCR_011812) and refined by manual inspection on the Geneious program (ver. 9) created by Biomatters (available from http://www.geneious.com/; RRID: SCR_010519). The leucine zipper domain of *Gryllus* Fra and Jra proteins, and the zinc finger motifs of *Gryllus* Egr protein were predicted by the SMART program (Schultz et al., 1998; Letunic et al., 2012; available from http://smart.embl-heidelberg.de; RRID: SCR_005026).

### Pharmacology

#### Induction of neuronal hyperexcitability

Adult male crickets 1 week after the imaginal molt were used for pharmacological experiment. Crickets were individually isolated in a 100-ml beaker (ø4.5 cm) for 3 days without food and water before pharmacological treatment. To induce neuronal hyperexcitability, 5 mM picrotoxin (PTX; Sigma-Aldrich; CAS: 124-87-8) dissolved in the cricket physiological saline (140 mM NaCl, 10 mM KCl, 1.6 mM CaCl_2_, 2 mM MgCl_2_, 44 mM glucose, 2 mM TES, pH 7.2) containing 5% dimethyl sulfoxide (DMSO) was injected. The cricket physiological saline containing 5% DMSO was injected as a vehicle control. To block *de novo* protein synthesis, 20 mM cycloheximide (Sigma-Aldrich; CAS: 66-81-9) dissolved in a cricket physiological saline was injected 1 h before PTX injection. 3 µl of each solution was injected into the head capsule using a 27-gauge needle attached to a 10-μl microsyringe (Hamilton 701 LT Syringe; Sigma-Aldrich).

#### Activation of intracellular signaling pathways

Adult male crickets 1 week after the imaginal molt were used for the pharmacological experiment. Crickets were individually isolated in a 100-ml beaker (ø4.5 cm) for 3 days without food and water before pharmacological treatment. To activate specific intracellular signaling pathways, 200 µM 12-*O*-tetradecanoylphorbol-13-acetate (TPA; Cayman Chemical; CAS: 16561-29-8), 1 mg/ml anisomycin (Cayman Chemical; CAS: 22862-76-6), 200 µM forskolin (Millipore; CAS: 66575-29-9), 10 mM S-nitroso-*N*-acetyl-DL-penicillamine (SNAP; Sigma-Aldrich; CAS: 67776-06-1), 200 µM A23187 (Sigma-Aldrich; CAS: 52665-69-7), which dissolved in the cricket physiological saline containing 5% DMSO, were injected. 3 µl of each solution was injected into the head capsule using a 27-gauge needle attached to a 10-μl microsyringe (Hamilton 701 LT Syringe; Sigma-Aldrich).

### Quantification of transcripts of the candidate IEGs

#### RNA extraction and reverse transcription for the initial expression analysis

Crickets were anesthetized on ice, and the brain (the supraesophageal and subesophageal ganglia without the optic lobes) was dissected in ice-cold saline, immediately chilled in liquid nitrogen, and stored at –80°C until use. Total RNA was extracted from a single cricket brain using the PureLink RNA Mini kit (Life Technologies) according to the manufacturer’s instruction. RNA samples were reverse transcribed using the High-Capacity cDNA Reverse Transcription Kit (Life Technologies). 0.5 µg of total RNA was reverse transcribed in a 20-µl reaction according to the manufacturer’s instruction.

#### RNA extraction and reverse transcription for the other RT-qPCR expression analyses

Total RNA was extracted from a single cricket brain using the TRIzol reagent (Life Technologies). Genomic DNA contamination was digested with DNase I (1 U in 10 µl reaction; TaKaRa) at 37°C for 1 h, followed by incubation at 75°C for 10 min to inactivate the enzyme. RNA samples were reverse transcribed using the High-Capacity cDNA Reverse Transcription Kit (Life Technologies). 1.5 µl of total RNA was reverse transcribed in a 10-µl reaction according to the manufacturer’s instruction.

#### Quantitative PCR

Quantitative PCR analysis was performed using the KAPA SYBR FAST qPCR Kit (Kapa Biosystems) and the Eco Real-Time PCR System (Illumina). 0.5 µl of each reverse transcription product was added to a 10 µl qPCR reaction. Quantitative PCR reaction was performed at 50°C for 2 min and 95°C for 5 min followed by 40 cycles of 95°C for 10 s and 60°C for 30 s each. Sequences of the primers used for qPCR are listed in Table 1.

**Figure 1.**
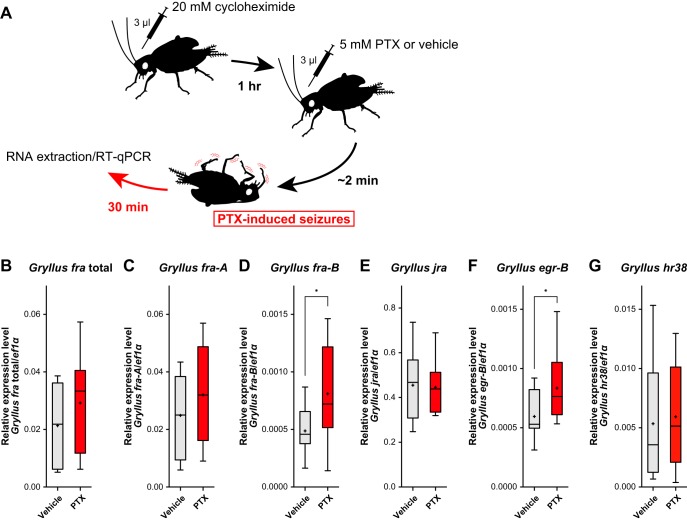
Expression of candidate neuronal IEGs in the brain of cycloheximide pretreated crickets 30 min after PTX injection. ***A***, PTX-induced neuronal hyperexcitability in the cricket. Crickets show seizure-like behavior ∼2 min after PTX injection. 1 h before PTX/vehicle injection, 20 mM cycloheximide was injected to block *de novo* protein synthesis. ***B–G***, Expression of (***B***) *Gryllus fra* total transcript, (***C***) *fra-A* isoform, (***D***) *fra-B* isoform, (***E***) *jra*, (***F***) *egr-B*, and (***G***) *hr38* in the brains of cycloheximide pretreated crickets 30 min after injection of vehicle (5% DMSO in saline) or PTX. Expression levels of each target gene were normalized with that of *Gryllus* *ef1α* gene (Fig. 1-1). RT-qPCR analyses were performed on eight biological replicates. Box plots indicate the 25th to 75th percentile ranges and central values. Error bars indicate the 5th to 95th percentile ranges. The “+” denotes the mean. Asterisks denote statistical significance (*, *p <* 0.05). See Table 3 for the details of statistical analysis. See Figs. 1-2, 1-3, 1-4, and 1-5 for the structures of the encoded proteins of candidate neuronal IEGs.

Gene expression levels were measured by the standard curve method using the EcoStudy Software (ver. 5.0; Illumina). Ten-fold serial dilutions of plasmids (pGEM-T Easy backbone) containing cDNA fragments of each target gene were used to plot standard curves. The template plasmid for *Gryllus fra* contained the isoform-specific sequence of *fra-A* (base 1-371 of LC215243) and the full-length cDNA of *Gryllus fra-B* (LC215244), which were tandemly inserted into the pGEM-T Easy. The template plasmid for *Gryllus jra* contained the full-length cDNA of *Gryllus jra* (LC215245). The template plasmid for *Gryllus egr* used for the initial RT-qPCR experiment (Fig. 1) contained the partial cDNA of *Gryllus egr-B* (base 1-429 of LC215246). The template plasmid for *Gryllus egr-B* and *EYFPnls:PEST* contained the partial cDNA of *Gryllus egr-B* (base 1–929 of LC215246) and the full-length coding sequence of *EYFP*, which were tandemly inserted into the pGEM-T Easy. The template plasmid for *Gryllus hr38* contained the partial cDNA sequence of *hr38* (base 1–374 of LC341255). The template plasmid for the pre-mRNAs of *Gryllus egr-B* and *hr38* contained the genomic fragment corresponding to the intron/exon boundary of *Gryllus egr* and *hr38* genes, respectively (base 1–437 of LC341288; base 1–260 of LC341256).

10.1523/ENEURO.0061-18.2018.f1-1 Figure 1-1 *Gryllus ef1α* is the most stable internal control for RT-qPCR analysis in the cricket brain. Validation of housekeeping genes as reference genes for RT-qPCR expression analysis in the cricket brain. ***A***, Expression levels of three housekeeping genes (*Gryllus β-actin*, *ef1α*, and *rpl32*) in the brain of wild-type adult crickets used for initial expression analysis (n = 16 including the PTX-injected and vehicle-injected crickets [n = 8, respectively]; see Fig. 1). The expression level of each housekeeping gene was normalized to the geometric mean of three housekeeping genes (GM*_β-actin_*_/_*_ef1α_*_/_*_rpl32_*; Vandesompele et al., 2002). Box plots indicate 25th to 75th percentile ranges and central values, and ‘+’ indicates mean. Error bars indicate 5th to 95th percentile ranges. In the cricket brain, *Gryllus β-actin* and *ef1α* genes were expressed at the same levels, but *Gryllus rpl32* was expressed more weakly than other two housekeeping genes. Among three housekeeping genes, the coefficient of variation (CV) of *Gryllus ef1α* was the lowest (CV of *Gryllus β-actin/*GM*_β-actin_*_/_*_ef1α_*_/_*_rpl32_*, 20.99%; CV of *Gryllus ef1α/*GM*_β-actin_*_/_*_ef1α_*_/_*_rpl32_*, 8.85%; CV of *Gryllus rpl32/*GM*_β-actin_*_/_*_ef1α_*_/_*_rpl32_*, 22.5%), indicating *Gryllus ef1α* is the most stably expressed in the cricket brain. ***A***, Gene expression stability of three housekeeping genes calculated using the Normfinder algorithm (Andersen et al., 2004). NormFinder algorithm supported that *Gryllus ef1α* is the most stable housekeeping gene (stability value of 0.036). The stability value of *Gryllus ef1α* was lower than that of the best combination pair (*Gryllus β-actin* and *ef1α*; stability value of 0.058). Download Figure 1-1, EPS file.

**Figure 2.**
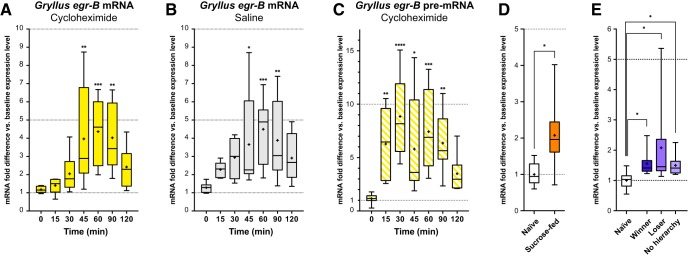
Expression characteristics of *Gryllus egr-B* in the cricket brain. ***A***, ***B***, Expression time course of *Gryllus egr-B* after PTX injection in the brains of (***A***) cycloheximide- and (***B***) saline-pretreated crickets. ***C***, Expression time course of *Gryllus egr-B* pre-mRNA in the brain of cycloheximide-pretreated crickets after PTX injection. ***D***, ***E***, Behaviorally evoked expression of *Gryllus egr-B* in the brain of crickets 1 h after (***D***) feeding of sucrose solution and (***E***) agonistic interaction. RT-qPCR analyses were performed on eight biological replicates. The expression levels were normalized to the mean of those of naïve animals (baseline expression level). Box plots indicate the 25th to 75th percentile ranges and central values. Error bars indicate the 5th to 95th percentile ranges. The “+” denotes the mean. Asterisks donate statistical significance to the control (0 min after PTX injection; ***A–C***) or to the naïve animals (***D***, ***E***; *, *p <* 0.05; **, *p <* 0.01; ***, *p <* 0.001; ****, *p <* 0.0001). See Table 3 for the details of statistical analysis. See Figs. 2-1, 2-2, and 1-5 for the expression characteristics of other candidate neuronal IEGs.

10.1523/ENEURO.0061-18.2018.f1-2 Figure 1-2 *Gryllus fra* gene encodes two protein isoforms closely related to insect and vertebrate Fos/Fra homologs. ***A***, Protein domain structures of *Gryllus* Fra proteins and Fos/Fra homologs of other species. Conserved domains and sequences important for transcriptional regulation are indicated by color boxes. DBD, DNA-binding domain; HOB, homology box; Leu zip, Leucine zipper domain; TBM, TATA-binding protein (TBP)-binding motif. *Gryllus fra-A* encodes a 382-amino-acid protein containing the DBD, Leu zip domains and the C-terminal regulatory domain. Vertebrate c-Fos proteins contain N-terminal and C-terminal transactivation domains and a repression domain at its C-terminus. The core motif of the N-terminal transactivation domain (HOB1-N) is well conserved among vertebrate Fra proteins, but not conserved in insect Fra proteins. Instead, insect Fra proteins contain sequence motifs which might play important roles in transactivation function: *Drosophila* Kayak-A isoform contains a δ-like motif (Ciapponi et al., 2001), and *Drosophila* Kayak-D/F isoforms contain glutamine-rich regions in the isoform-specific N-terminal region. *Gryllus* Fra-A isoform and its related insect Fra proteins contain an acidic patch structurally resembles the acidic activation domains of many eukaryotic transcriptional activators such as Gal4, VP16, p53 and EcR-B1 (Cress and Triezenberg, 1991; Ruden, 1992; Regier et al., 1993; Watanabe et al., 2010). In addition, the T/P-rich region is conserved in most insect Fra proteins. *Gryllus fra-B* encodes an N-terminal truncated 284-amino-acid protein. ***B***, Sequence alignment of the N-terminal region of *Gryllus* Fra-A and insect Fra/Kayak isoforms, and the C-terminal regulatory domain of Fos/Fra proteins. The conserved residues are marked with asterisks above the alignments. The amino acid residues are represented in the default color scheme of ClustalX. Positions of conserved domains/motifs were indicated by bars under the alignments. GenBank IDs of proteins are following: *Apis mellifera* Kayak-X1, XP_006564216; *Bombyx mori* Kayak-X2, XP_004921825; *Drosophila melanogaster* Kayak-A, NP_001027579; *D. melanogaster* Kayak-B, NP_001027578; *D. melanogaster* Kayak-D, NP_001027580; *D. melanogaster* Kayak-F, NP_001027577; *Homo sapience* c-Fos, NP_005243; *H. sapience* Fra1, NP_005429: *H. sapience* Fra2, NP_005244; *H. sapience* Fos-B, NP_006723; *Tribolium castaneum* Kayak-C, NP_001164294. Download Figure 1-2, EPS file.

**Figure 3.**
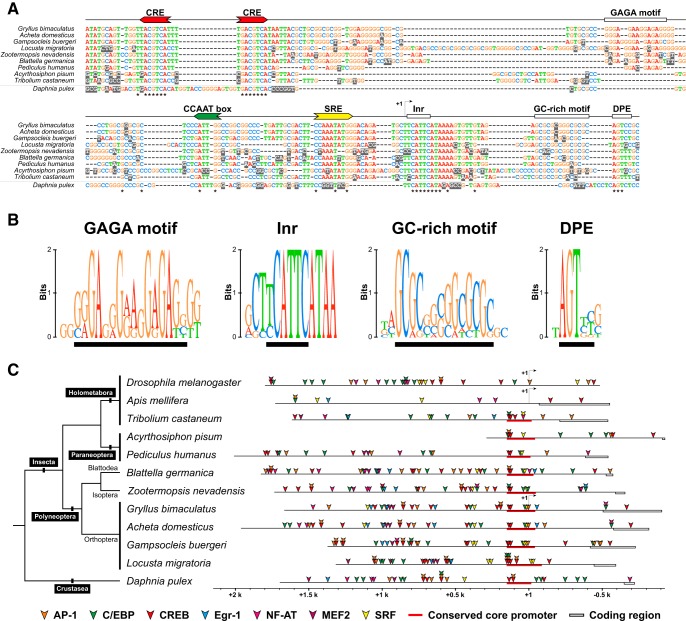
Gene regulatory regions of the insect/crustacean *egr-B* homologs. ***A***, Putative core promoter regions of basal insect and crustacean *egr-B* homologs share a high-level sequence similarity. The upstream sequences of insect/crustacean *egr-B* homologs are aligned with the core promoter region of *Gryllus egr-B*. The conserved bases are marked with asterisks under the alignment. *Cis*-regulatory elements and sequence motifs that are conserved are indicated above the alignment. CRE, cAMP-responsive element; SRE, serum response element; Inr, initiator element; DPE, downstream promoter element. ***B***, Sequence logo representation of the conserved motifs in the core promoter region of insect *egr-B* homologs. The sequence logo of the GAGA motif was generated by multiple alignment of the upstream sequences of polyneopteran *egr-B* homologs. The other sequence logos were generated by multiple alignment of the upstream sequences of insect *egr-B* homologs. The positions of conserved motifs are indicated by black bars under the logo. ***C***, Schematic representation of the gene regulatory regions of insect/crustacean *egr-B* homologs. The genomic regions were aligned to the position of the +1 site of *Gryllus egr-B* or the 5′-end of the putative core promoter region. The red bars indicate genomic regions aligned in Fig. 3*A*. Positions of transcription factor binding sites predicted using the LASAGNA-Search 2.0 program (score >8.0) are indicated by arrowheads. The phylogenetic relationship of insect/crustacean species is indicated as a phylogram tree. AP-1, activator protein 1; CREB, cAMP response element-binding protein; C/EBP, CCAAT-enhancer-binding protein; MEF2, myocyte enhancer factor 2; NF-AT, nuclear factor of activated T-cells; SRF, serum response factor. See Table 2 for the details of genomic sequences used for promoter analysis. See Fig. 3-1 and Table 3-1 for the structural conservations of the transcription factors used for the binding site prediction.

10.1523/ENEURO.0061-18.2018.f1-3 Figure 1-3 *Gryllus jra* gene encodes a protein closely related to insect and vertebrate Jun/Jra homologs. ***A***, Protein domain structures of *Gryllus* Jra protein and Jun/Jra homologs of other species. Conserved domains and sequences important for transcriptional regulation are indicated by color boxes. DBD, DNA-binding domain; HOB; homology box; Leu zip, Leucine zipper domain. ***B***, Sequence alignment of the conserved domains for transcriptional regulation (δ domain and HOB motifs). The conserved residues are marked with asterisks above the alignments. The amino acid residues are represented in the default color scheme of ClustalX. Positions of conserved domains/motifs were indicated by bars under the alignment. GenBank IDs of proteins are following: *D. melanogaster* Jra, NP_476586; *H. sapience* c-Jun, NP_002219; *H. sapience* Jun-B, NP_002220; *H. sapience* Jun-D, NP_005345. Download Figure 1-3, EPS file.

**Figure 4.**
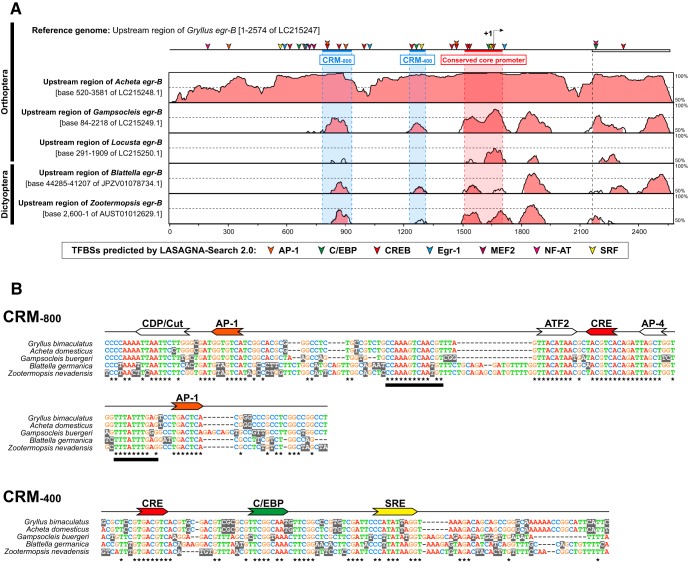
Phylogenetic footprinting revealed conserved *cis*-regulatory modules in the upstream regions of polyneopteran *egr-B* homologs. ***A***, mVISTA plot of the upstream regions of polyneopteran *egr-B* homologs based on MLAGAN alignment using the upstream region of *Gryllus egr-B* as a reference sequence. Positions of potential transcription factor binding sites in the upstream region of *Gryllus egr-B* are indicated by arrowheads (Fig. 3*B*). The horizontal and vertical axes of the plot represent the position in the sequences and the percentage identity, respectively. Two conserved *cis*-regulatory modules (CRMs; CRM_-800_ and CRM_-400_) and the conserved core promoter region are shaded blue and red on the plot, respectively. ***B***, Nucleotide sequence alignments of two conserved CRMs (CRM_-800_ and CRM_-400_) found in the upstream region of polyneopteran *egr-B* homologs. The conserved bases are marked with asterisks under the alignment. *Cis*-regulatory elements conserved among most of the sequences are indicated above the alignment. Black bars under the alignments indicate sequence motifs conserved across species where no transcription factor is assigned. AP-1, binding site for activator protein 1; AP-4, binding site for activating enhancer binding protein 4; ATF2, binding site for activating transcription factor 2; CDP/Cut, binding site for CCAAT-displacement protein/cut homeobox; C/EBP, binding site for C/EBP; CRE, cAMP-responsive element; SRE, serum response element. See Table 2 for the details of genomic sequences used for promoter analysis.

10.1523/ENEURO.0061-18.2018.f1-4 Figure 1-4 *Gryllus egr* gene encodes a protein closely related to insect and vertebrate Egr-1 homologs. ***A***, Protein domain structures of *Gryllus* Egr-B protein and Egr homologs of other species. Conserved domains and sequences important for transcriptional regulation are indicated by color boxes. Three C_2_H_2_-type zinc finger domains, as well as a nuclear localization signal (NLS) and a potential acetylation site (Ac), were highly conserved across vertebrate and invertebrate Egr homologs. On the other hand, we found low sequence conservation in the repressor domain between Egr homologs of insect and other species. The WW binding motif (PPxY, where x = any amino acid), which involved in protein–protein interaction with the Yes kinase-associated protein 1 (Zagurovskaya et al., 2009), was conserved across vertebrate and invertebrate Egr homologs. ***B***, Sequence alignment of the C-terminal region of Egr proteins. The conserved residues are marked with asterisks above the alignment. Positions of functional domains important for DNA-binding, protein localization, and transcriptional regulation are indicated by bars under the alignment. ***C***, Sequence alignment of the N-terminal region of insect/crustacean Egr-B proteins. The N-termini of insect/crustacean Egr-B proteins were highly conserved (conserved N-terminal motif 1; consensus sequence: MIM(D/E)FΨ(D/E)TL, where Ψ = bulky hydrophobic residues). Another conserved motif was found at residues from 105 to 140 of *Gryllus* Egr (conserved N-terminal motif 2). These two conserved motifs were only found in the N-terminal region of the insect/crustacean Egr-B proteins, but not in the vertebrate Egr homologs. Another Egr isoform (Egr-A or Stripe-A) found in several insect species (e.g. fruit flies and honeybees) contains an N-terminal extension with polyglutamine stretch (Ugajin et al., 2016). The conserved residues are marked with asterisks above the alignment. The amino acid residues are represented in the default color scheme of ClustalX. The positions of conserved motifs were indicated by bars under the alignment. GenBank IDs of proteins are following: *Acyrthosiphon pisum* Egr, XP_001943786; *Anoplophora glabripennis* Egr-B, XP_018579268; *Apis mellifera* Egr-B (*Am*Egr variant III), ANS58852; *Aplysia californica* Egr-1-like, NP_001268725; *Calliphora vicina* Stripe-B, AAZ95459; *D. melanogaster* Stripe-B, NP_732289; *Tribolium castaneum* Egr, XP_015837968l; *Mus musculus* Egr-1, NP_031939. The N-terminal sequences of *Homarus americanus* Egr-B, *Periplaneta americana* Egr-B, and *Procambarus clarkii* Egr-B were deduced from following transcriptome shotgun assembly sequences: GEBG01017003.1, GEIF01013459.1, and GBEV01045599.1, respectively. Download Figure 1-4, EPS file.

**Figure 5.**
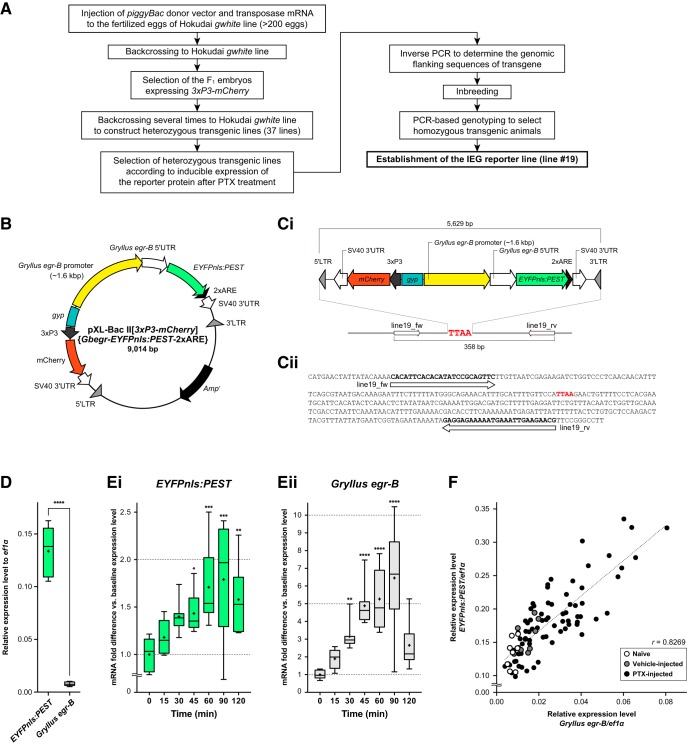
IEG promoter-driven transgenic reporter system in the cricket brain. ***A***, Flowchart of the experimental procedures to establish the IEG reporter line. See the Materials and Methods section for detail. ***B***, Schematic representation of the *piggyBac* transgenic vector for the IEG promoter-driven transgenic reporter system. The vector harbors the expression cassette of *EYFPnls:PEST* driven by the *Gryllus egr-B* promoter. *3xP3-mCherry* was used as a visible selection marker. A *gypsy* insulator sequence (*gyp*) was inserted between two expression cassettes. ARE, AU-rich element; LTR, long terminal repeat. ***Ci***, Schematic representation of the *piggyBac* insertion in the IEG reporter line. A 5629-bp insertion was inserted into the *piggyBac* donor TTAA site (highlighted in red). To conduct genotyping PCR, two primers, line19_fw and line19_rv, were designed at the 5′ and 3′ flanking region of the insertion sites, respectively. ***Cii***, The nucleotide sequence of the genomic region flanking the *piggyBac* insertion in the IEG reporter line. The *piggyBac* donor TTAA site is highlighted in red. The positions of the annealing site of primers for genotyping PCR are indicated by white arrows under the sequence. ***D***, Basal mRNA expressions of *EYFPnls:PEST* and *Gryllus egr-B* in the brain of naïve IEG reporter line. ***E***, Expression time course of (***Ei***) *EYFPnls:PEST* and (***Eii***) *Gryllus egr-B* in the brain of the IEG reporter line after PTX injection. RT-qPCR analyses were performed on eight biological replicates. The expression levels were normalized to the mean of those of naïve animals (baseline expression level). Box plots indicate the 25th to 75th percentile ranges and central values. Error bars indicate the 5th to 95th percentile ranges. The “+” denotes the mean. Asterisks donate statistical significance to the control (naïve animals; *, *p <* 0.05; **, *p <* 0.01; ***, *p <* 0.001; ****, *p <* 0.0001). ***F***, Correlation plot between the expression levels of *EYFPnls:PEST* and *Gryllus egr-B* in the brains of the IEG reporter line. The data from PTX-injected crickets (*n* = 75; black circles), vehicle pre-injected crickets (*n* = 8; gray circles), and naïve crickets (*n* = 8; white circles) were plotted. See Table 3 for the details of statistical analysis.

10.1523/ENEURO.0061-18.2018.f1-5 Figure 1-5 Molecular cloning and expression characteristics of *Gryllus hr38* in the cricket brain. ***A***, Comparison of the amino acid sequences of *Gryllus* hr38 deduced from its partial cDNA and its corresponding part of hr38 homologs in other insects. The conserved residues are marked with asterisks above the alignments. The amino acid residues are represented in the default color scheme of ClustalX. The domain structure of *Drosophila* DHR38-D is represented above the alignment. GenBank IDs of proteins are following: *D. melanogaster* DHR38 isoform D (DHR38-D), NP_001163024; *Bombyx mori* HR38, P49870.1; *Tribolium castaneum* HR38, XP_008194320.1; *Apis mellifera* HR38, XP_016773251.1; *Zootermopsis nevadensis* HR38, KDR09534.1. ***B***, ***C***, Expression of *Gryllus hr38* after PTX injection in the brains of (***B***) cycloheximide- and (***C***) saline-pretreated crickets. Asterisks indicate statistical significance to control (0 min after PTX injection). In both pretreatment groups, *Gryllus hr38* reached a maximum 60–90 min after PTX injection (∼60-fold and ∼15-fold up-regulation relative to the naïve animals and to the control (0 min after PTX injection), respectively) and remained at a high level 120 min after PTX injection. The expression kinetics of *Gryllus hr38* was not affected by blockade of *de novo* protein synthesis (two-way ANOVA, Effect of pre-treatment: *F*(1,98) = 0.2142, *p* = 0.6445; Effect of time: *F*(6,98) = 14.45, *p* < 0.0001; interaction: *F*(6,98) = 0.5547, *p* = 0.7652). ***D***, Expression time course of the pre-mRNA of *Gryllus hr38* in the brain of cycloheximide- and saline-pretreated crickets after PTX-injection. Asterisks and daggers indicate statistical significance to control (0 min after PTX injection) within each pre-treatment group (***, *p* < 0.001; ††††, *p* < 0.0001). In both pretreatment groups, the expression of *Gryllus hr38* pre-mRNA was significantly elevated 30 min after PTX injection (200∼300-fold up-regulation relative to the naïve animals), and rapidly decreased to near baseline level by 60–90 min after injection. The expression kinetics of *Gryllus hr38* pre-mRNA was not affected by blockade of *de novo* protein synthesis (two-way ANOVA, Effect of pre-treatment: *F*(1,98) = 0.5631, *p* = 0.4548; Effect of time: *F*(6,98) = 16.11, *p* < 0.0001; interaction: *F*(6,98) = 1.733, *p* = 0.1212). ***E***, Correlation plot between the expression levels of *Gryllus hr38* and *Gryllus egr-B* in the brains of cycloheximide-pretreated crickets. The data from the cycloheximide- and PTX-injected crickets (n = 48; black circles), cycloheximide pretreated crickets (n = 8; gray circles), and naïve crickets (n = 8; white circles) were plotted. ***F***, ***G***, Behaviorally evoked expression of *Gryllus hr38* in the brain of crickets 1 h after (***F***) sucrose feeding and (***G***) agonistic interaction. The expression levels were normalized to the mean of those of the naïve animals (baseline expression level). An asterisk indicates statistical significance between the indicated groups. RT-qPCR analyses were performed on eight biological replicates. Box plots indicate the 25th to 75th percentile ranges and central values. Error bars indicate the 5th to 95th percentile ranges. The “+” denotes the mean. Asterisks donate statistical significance to the control (0 min after PTX injection; ***B***, ***C***) or to the naïve animals (***F***, ***G***; *, *p* < 0.05; **, *p* < 0.01; ***, *p* < 0.001; ****, *p* < 0.0001). See Table 3 for the details of statistical analysis.Download Figure 1-5, EPS file

Expression levels of each target gene were normalized with that of *Gryllus* *ef1α* gene, which was selected from three housekeeping genes (*Gryllus β-actin*, *ef1α*, and *rpl32*) according to expression stability in the brain (Fig. 1-1). The template plasmids for *Gryllus β-actin*, *ef1α*, and *rpl32* contained the full-length cDNA of the genes (AB626808.1, AB583232.1, and AB626807.1), respectively.

### Determination of the transcription start sites (TSSs) of *Gryllus* and *Apis egr-B* homologs

We performed the 5′ RACE using the FirstChoice RLM-RACE kit (Ambion) to clone the 5′ ends of *Gryllus* and *Apis egr-B* homologs. RNA processing reactions were conducted according to the manufacturer’s instruction. Reverse transcription was conducted using the Superscript III reverse transcriptase (Life Technologies) according to the manufacturer’s instruction. 5′ RACE PCRs were conducted using the Q5 High-Fidelity DNA polymerase (New England Biolabs). Complementary DNAs were inserted into the pGEM-T Easy vector (Promega) by standard TA cloning procedure using the T4 DNA ligase or the Gibson assembly technique using the Gibson Assembly Master Mix (New England Biolabs). Complementary DNA fragments for the Gibson assembly cloning were amplified with primers with 5′ extensions (5′-GCC GCG GGA ATT CGA TT-3′ was attached to the 5′ end of the 5′ RACE inner primer; 5′-CCG CGA ATT CAC TAG TGA TT-3′ was attached to the 5′ end of the gene-specific reverse primer). More than 20 cDNA clones were sequenced to determine the TSSs.

### Isolation of the upstream regions of orthopteran *egr-B* homologs

To isolate the putative core promoter region, genomic DNA of four orthopteran insects were extracted using the Wizard Genomic DNA Purification Kit (Promega). Based on the high-level sequence similarity in the core promoter region of *egr* homologs in basal insects, we designed primers to amplify the putative core promoter region of orthopteran *egr-B* homologs. PCRs were performed using a pair of the forward and reverse primers listed in Table 1. Then, the upstream regions of orthopteran *egr-B* homologs were obtained by using inverse PCR. The procedure for inverse PCR was modified from Watanabe and Aonuma (2014). Genomic DNAs of three orthopteran insects were digested with BfaI, BglII, DpnII, EaeI, HhaI, HindIII, MseI, MspI, NlaIII, TaqαI, XapI or XceI. 10 ng of restriction fragments were circularized using the T4 DNA ligase (Thermo Fisher Scientific) in 10 µl reaction. Primers were designed on the basis of the nucleotide sequence of the putative core promoter of *egr-B* homologs. Inverse PCRs were repeatedly performed to obtain >1.5-kbp genomic DNA fragments flanking the first exon of *egr-B* homologs. All PCRs were conducted using the Q5 High-Fidelity DNA Polymerase (New England Biolabs). PCR products were cloned into the pGEM-T Easy vector (Promega) and their sequences were determined.

### Database search and sequence comparison of stimulus-regulated transcription factors among vertebrates and insects

To understand the structural features of the upstream sequence of insect/crustacean *egr-B* homologs, we searched potential binding sites for stimulus-regulated transcription factors (TFs) conserved among vertebrates, insects, and crustaceans. There have been few reports on the DNA-binding specificities of stimulus-regulated TFs in insects and crustaceans, so we examined sequence conservation in the DNA-binding domains of stimulus-regulated TFs between mouse and insects, to assess whether each candidate TF in insects might have DNA-binding properties similar to vertebrate homologs. We analyzed components of the stimulus-regulated transcription factor complexes which are known to involved in regulating stimulus-regulated gene expression (Dolmetsch et al., 1997; Hu et al., 2001; Roy et al., 2002; Tullai et al., 2007) as follows: AP-1, CREB, C/EBP, Egr, NFAT, MEF2, and SRF, which include Fos-family proteins, Jun-family proteins, ATF/CREB family proteins, Maf-family proteins, C/EBPs, Egr-family proteins, NFATs, MEF2, and SRF. We first retrieved mouse homologs of stimulus-regulated TFs from the GenBank protein sequence database. Then, BLASTp searches were conducted in search for homologs of stimulus-regulated TFs in *Drosophila melanogaster*, *Tribolium castaneum*, *Apis mellifera*, *Acyrthosiphon pisum*, and *Zootermopsis nevadensis.* We also performed tBLASTn searches on the *Gryllus firmus* transcriptome shotgun assembly (TSA) database to find cDNA sequence of the cricket homologs of the target genes. The amino acid sequences of the DNA binding domains of stimulus-regulated TFs of mouse and insects were aligned using the MAFFT or MUSCLE algorithms on the Geneious program (ver. 9). GenBank IDs of stimulus-regulated TFs are listed in Table 3-1.

### Structural analysis of the potential gene regulatory region of *egr* genes

Potential transcriptional regulatory sequences in the upstream sequences of insect/crustacean *egr-B* homologs were predicted using the LASAGNA-Search 2.0 program (Lee and Huang, 2014; available from http://biogrid-lasagna.engr.uconn.edu/lasagna_search/; RRID: SCR_010883). Potential binding sites for stimulus-regulated transcription factors common to vertebrates and insects were searched. Phylogenetic footprinting analysis of the upstream regions of the polyneopteran *egr-B* homologs was conducted using the mVISTA program (Frazer et al., 2004; available from http://genome.lbl.gov/vista/mvista/submit.shtml) with default setting. GenBank IDs of genomic sequences are listed in Table 2.

**Table 2.** Genomic sequences used for promoter analysis

Species	GenBank ID	Genomic region targeted for TFBS prediction	Position of the conserved core promoter	Position of the +1 site	Position of CRM_-800_	Position of CRM_-400_
*Drosophila melanogaster*	NT_033777.2	Base 13945983 to 13948265		Base 13947783		
*Apis mellifera*	NC_007084.3	Base 6690515 to 6692794		Base 6691064**		
*Tribolium castaneum*	NC_007417.3	Base 2995987 to 2998141	Base 2997452 to 2997620(169 bp)			
*Acyrthosiphon pisum*	ABLF02030506.1	Base 25286 to 24071	Base 25155 to 24964(192 bp)			
*Pediculus humanus*	NW_002987224.1	Base 14669 to 12120	Base 12809 to 12649(161 bp)			
*Blattella germanica*	JPZV01078734.1	Base 44,303 to 41,904	Base 42626 to 42446(181 bp)		Base 43369 to 43205 (165 bp)	Base 42891 to 42809 (83 bp)
*Zootermopsis nevadensis*	AUST01012629.1	Base 2539 to 150	Base 954 to 753 (197 bp)		Base 1667 to 1493 (83 bp)	Base 1248 to 1170 (79 bp)
*Gryllus bimaculatus*	LC215247*	Base 1 to 2574	Base 1518 to 1711 (194 bp)	Base 1668**	Base 786 to 937 (152 bp)	Base 1235 to 1316 (82 bp)
*Acheta domesticus*	LC215248*	Base 1 to 2781	Base 2113 to 2306 (194 bp)		Base 1358 to 1509 (152 bp)	Base 1830 to 1911 (82 bp)
*Gampsocleis buergeri*	LC215249*	Base 1 to 2098	Base 1223 to 1413 (191 bp)		Base 498 to 652 (155 bp)	Base 945 to 1024 (80 bp)
*Locusta migratoria*	LC215250*	Base 1 to 1909	Base 1166 to 1404 (239 bp)			
*Daphnia pulex*	ACJG01000376.1	Base 1585904 to 1583495	Base 1584357 to 1584191(167 bp)			

* Genomic sequences were determined in this study.

** Positions of the TSS(s) were determined in this study.

### Construction of *piggyBac* donor plasmid

A *piggyBac* donor plasmid pXL-BacII[*3xP3-mCherry*]{*Gbegr*-*EYFPnls:PEST-2xARE*} was constructed to examine promoter activity of *Gryllus egr-B in vivo*. This plasmid contains the expression cassettes for an IEG reporter (*Gbegr*-*EYFPnls:PEST-2xARE*) and a visible selection marker (*3xP3-mCherry*). The IEG reporter cassette consists of (1) a 2.2-kb upstream sequence of *Gryllus* e*gr-B* coding sequence; (2) a coding sequence of nuclear-targeted EYFP C-terminally fused to a PEST domain of mouse ornithine decarboxylase (EYFPnls:PEST); (3) two repeats of synthetic AU-rich elements (2xARE); and (4) a SV40 3′ untranslated region (UTR). The expression cassettes of the IEG reporter and *3xP3-mCherry* were separated by the *gypsy* insulator sequence. The donor plasmid was constructed through the following procedures.

#### Gbegr-EYFPnls:PEST-2xARE expression cassette

A ∼2.2-kb genomic fragment upstream to *Gryllus* e*gr-B* was amplified by PCR from *Gryllus bimaculatus* genomic DNA purified using the Wizard Genomic DNA Purification Kit (Promega). A nucleotide sequence encoding the nuclear localization signal of the SV40 Large T-antigen (PKKKRKV) was added to the 3′ end of the coding sequence of EYFP derived from the pBSII-ITR1.1k-EYFP plasmid by PCR. The coding sequence of the PEST domain of mouse ornithine decarboxylase (residues 416–461 of NP_038642.2) was synthesized by Eurofins Genomics. Two repeats of synthetic AU-rich elements (5′-TTT ATT TAT TTA TTT ATT TA-3′) were added to the 5′ end of the SV40 3′ UTR derived from the pBSII-ITR1.1k-EYFP plasmid by PCR. The DNA fragments of the promoter, protein coding sequence, and 3′ UTR were assembled in order by restriction enzyme digestion and ligation, or by using the Gibson assembly technique.

#### 3xP3-mCherry expression cassette

The *mCherry* coding sequence derived from the pTRE3G-mCherry vector (TaKaRa) was inserted into the NcoI/XbaI-digested pBSII-ITR1.1k-EYFP plasmid.

#### Plasmid construction

First, a *gypsy* insulator sequence derived from the pGreen-Pelican plasmid (DGRC stock number: 1015) was inserted between the HindIII and SphI sites of the pXL-BacII plasmid. Then, the *Gbegr*-*EYFPnls:PEST-2xARE* expression cassette was inserted between the SphI and XbaI sites of the vector to construct pXL-BacII{*Gbegr*-*EYFPnls:PEST-2xARE*}. Finally, the *3xP3*-*mCherry* expression cassette (*3xP3-mCherry-SV40 3′UTR*) was inserted into the HindIII-digested pXL-BacII{*Gbegr*-*EYFPnls:PEST-2xARE*} using the Gibson assembly technique.

All PCRs were conducted using the Q5 High-Fidelity DNA Polymerase (New England Biolabs). Gibson assembly was performed using the Gibson Assembly Master Mix (New England Biolabs). After construction, the plasmid was amplified in *Escherichia coli* strain DH5ɑ and purified using the PureLink HiPure Plasmid Kit (Life technologies).

### *piggyBac* transposon-mediated transgenesis

#### In vitro synthesis of piggyBac transposase mRNA

To construct the expression plasmid for *piggyBac* transposase mRNA (pTD1-*piggyBac*), the coding sequence of *piggyBac* transposase derived from the pBSII-IFP2-orf plasmid was inserted into the pTD1 expression vector (Shimadzu). The expression cassette of the *piggyBac* transposase in the pTD1-*piggyBac* was amplified by PCR with the Q5 High-Fidelity DNA Polymerase (New England Biolabs) with following primers (forward 5′-GCA GAT TGT ACT GAG AGT G-3′ and reverse 5′-CAG GAA ACA GCT ATG AC-3′). The PCR fragment was subsequently used as templates for *in vitro* transcription using the T7 mScript Standard mRNA Production System (CELLSCRIPT). Then, the 5′ cap and poly(A) tail were added to the transcribed RNA using the kit. The *piggyBac* mRNA was purified by phenol-chloroform extraction and concentrated by ammonium acetate precipitation.

#### Microinjection

The *piggyBac* donor plasmid (1 µg/μl) and *piggyBac* mRNA (1 µg/μl) were dissolved in nuclease-free water (Qiagen) to make an injection solution for germline transformation. The injection solution was injected into fertilized eggs of the Hokudai *gwhite* strain. Eggs were laid in a wet cotton or paper towel and collected within 30 min of egg laying. They were rinsed with 70% ethanol, aligned on the hand-made injection chamber, shortly air-dried, and covered with mineral oil (Nacalai Tesque). Micropipettes were pulled on a micropipette puller (PA-81; Narishige) using thin-walled filament glass capillary (GD-1.2; Narishige). The capillaries were backfilled with the injection solution using the GELoader tip (Eppendorf) and connected to the IM-6 microinjector (Narishige) filled with mineral oil. Injection solution was injected into the dorsal posterior part of the eggs. Eggs were transferred onto the wet cotton in the glass Petri dish immediately after injection. Petri dishes were incubated at 28°C in the moist chamber until hatching.

#### Genetics

Embryos injected with the *piggyBac* donor plasmid and *piggyBac* mRNA were raised to adult and backcrossed to Hokudai *gwhite* strain. The *F_1_* embryos exhibiting mCherry fluorescence in the compound eyes were selected. Each transgenic cricket was repeatedly backcrossed to the Hokudai *gwhite* strain to generate heterozygous lines.

### Genotyping

To establish IEG promoter-driven reporter lines, heterozygous transgenic lines were selected according to the inducible expression of EYFPnls:PEST protein after PTX treatment. 6 h after PTX injection, the brains were dissected for EYFP immunohistochemistry according to the experimental procedure described below. Then, the genomic flanking sequence of *piggyBac* insertion was determined by inverse PCR. To establish homozygous lines, each heterozygous line was inbred, and homozygous transgenic crickets were selected by PCR-based genotyping.

#### Inverse PCR

First, the genomic regions flanking the *piggyBac* insertions were amplified by inverse PCR. Genomic DNA of the heterozygous transgenic crickets was extracted using the Wizard Genomic DNA Purification Kit (Promega). Genomic DNA was digested with DpnII, HindIII, or MspI. 10 ng of restriction fragments were circularized using the T4 DNA ligase (Promega) in 10 µl reaction. PCRs were performed to amplify the 5′ and 3′ flanking regions of the *piggyBac* insertion using the Q5 High-Fidelity DNA Polymerase (New England Biolabs). Following primers were used for amplification of the 5′ flanking region of the insertion (primers for 1st PCR: 3′-GCT CCA AGC GGC GAC TGA GAT GTC C-3′ and 5′-GCT TGT CAA TGC GGT AAG TGT CAC TG-3′; primers for nested PCR: 5′-GAC GGA TTC GCG CTA TTT AGA AAG AGA G-3′ and 5′-CGG TAA GTG TCA CTG ATT TTG AAC TAT AAC G-3′). The 3′ flanking region of the insertion was amplified by inverse PCR using primers designed on the basis of the nucleotide sequence of the 5′ flanking region of the insertion. PCR products were cloned into the pGEM-T Easy vector (Promega) and their sequences were determined.

#### PCR-based genotyping

Heterozygous transgenic lines were inbred to generate homozygous transgenic animals. Each adult transgenic cricket in the inbred colonies was isolated after the imaginal molt, and homozygous animals were selected by PCR-based genotyping. Genomic DNA was isolated from the wings of newly emerged transgenic crickets using the modified HotSHOT method (Meeker et al., 2007). The tip of a hind wing (∼2 mg) was dissected from crickets and homogenized in liquid nitrogen. Samples were incubated at 95°C for 30–60 min in 50 µl of 50 mM NaOH. 5 µl of 1 M Tris-HCl (pH 7.5) was added to each tube for neutralization. After brief centrifugation, supernatants were diluted 16 times with TE buffer (pH 8.0). PCR was conducted using the Q5 High-Fidelity DNA Polymerase (New England Biolabs) in a 10-µl reaction. 0.5 µl of genomic DNA solutions were added to the reactions. For genotyping, forward and reverse primers were designed at the 5′ and 3′ genomic regions flanking the *piggyBac* insertion (forward primer [line19_fw primer], 5′-CAC ATT CAC ACA TAT CCG CAG TTC-3′; reverse primer [line19_rv primer], 5′-CGT TCT TCA ATT TCA TTT TTC TCC TC-3′), respectively (Fig. 5*C*). PCR products were run through 1.5% agarose gel in TBE buffer and visualized by ethidium bromide. We selected homozygous transgenic animals as follows: a 358-bp genomic fragment was amplified if the cricket had no transgene or one copy of transgene (heterozygous crickets), whereas no PCR product was amplified from homozygous crickets.

### Generation of anti-*Gryllus* Tdc2 polyclonal antibody

#### Complementary DNA cloning for tyrosine decarboxylase (tdc) genes

The full-length cDNA of two *tdc* genes (*Gryllus tdc1* and *tdc2*) was obtained as follows. A partial cDNA of *Gryllus tdc1* was amplified using GSPs designed on the basis of a *G. bimaculatus* EST clone corresponding to *Gryllus tdc1* (GenBank ID; AK258423). A partial cDNA of *Gryllus tdc2* was amplified using degenerate primers designed on the basis of conserved amino acid sequences (IEYADSFNT and FFVRMVSDP) among the insect Tdc2 proteins. Then, 5′ and 3′ RACEs were performed as described above to determine translational initiation and termination sites of the genes. Finally, full-length cDNAs of the genes were amplified, and their nucleotide sequences were determined. Sequences of the primers are listed in Table 1.

#### Recombinant protein expression and antibody production

A cDNA fragment encoding amino acids 470–683 of *Gryllus* Tdc2 protein (GenBank ID: BAO52000.1) was inserted between the BamHI and EcoRI sites of the pGEX-6P-1 vector (GE Health care Japan). The antigen peptide was produced as a glutathione S-transferase (GST) fusion protein in the Rossetta 2(DE3)pLysS cells (Millipore). GST-tagged protein was bound to the Glutathione HiCap Matrix (Qiagen), and antigen peptide was cleaved using the PreScission protease (GE Health Care Japan). The anti-*Gryllus* Tdc2 polyclonal antibody was generated in the guinea pig, and IgG fraction was purified from antiserum with Protein A (Frontier Institute; Ishikari-shi). Antibody specificity was checked by Western blot. An intensely stained band of ∼200 kDa was detected in the lane of the central brain, which matches the calculated molecular mass of *Gryllus* Tdc2 protein (data not shown). Anti-*Gryllus* Tdc2 polyclonal antibody (1.4 mg IgG/ml) was stored in 50% glycerol at –20°C.

### Whole-mount immunohistochemistry

Whole-mount fluorescent immunohistochemistry was performed according to Gonzalez-Bellido and Wardill (2012) with modifications. Crickets were anesthetized on ice, and the brain (the supraesophageal and subesophageal ganglia without the optic lobes) was dissected in ice-cold saline. The brains were fixed with 4% paraformaldehyde (PFA) in phosphate-buffered saline (PBS; P-5493, Sigma-Aldrich) at 4°C overnight. When we conducted anti-octopamine immunohistochemistry, brains were fixed with 1% glutaraldehyde in 0.1 M sodium cacodylate buffer (pH 7.4; Electron Microscopy Sciences) with 1% sodium metabisulfite (SMB) for 2 h on ice, and with 4% PFA in PBS at 4°C overnight. The samples were treated with 0.5% sodium borohydride in Tris-buffered saline (TBS; 0.05 M Tris-HCl, 77 mM NaCl, pH 7.4) containing 0.45% SMB for 1 h.

After three 30-min rinses with PBS, the brains were incubated with 500 µg/ml collagenase/dispase (Roche Applied Science) and 300 µg/ml hyaluronidase (Sigma-Aldrich) in PBS at 37°C for 1 h. After three 15-min rinses with PBS containing 0.5% Triton X-100 (PBS-Tx), the brains were blocked with 5% normal goat serum in PBS-Tx (blocking solution) at 4°C overnight. Then, the brains were incubated with the primary antibodies in the blocking solution at 4°C for 3–4 days. The reporter protein was detected by the chicken anti-GFP IgY (1:1000; Aves Labs, Cat# GFP-1020; RRID: AB_10000240) or the rabbit anti-GFP pAb (1:1000; Medical & Biological Laboratories, Cat# 598S; RRID: AB_591816). The DUM neurons were labeled with the guinea pig anti-*Gryllus* Tdc2 antibody (1:1000) and/or the mouse monoclonal anti-octopamine antibody (1:1000; Jena Bioscience, Cat# ABD-029, RRID: AB_2315000). After five 30-min rinses in PBS-Tx, the brains were incubated with secondary antibodies in the blocking solution at 4°C for 3 days. The Cy3-conjugated goat anti-chicken IgY (1:400; Jackson ImmunoResearch, Cat# 103-165-155; RRID: AB_2337386) and the Cy2-conjugated goat anti-rabbit IgG (1:400; Jackson ImmunoResearch, Cat# 111-225-144; RRID: AB_2338021) were used to detect the reporter protein. The Cy3-conjugated donkey anti–guinea pig IgG antibody (1:400; Jackson ImmunoResearch, Cat# 706-165-148; RRID: AB_2340460) and the Cy2-conjugated goat anti-mouse IgG antibody (1:100; Jackson ImmunoResearch, Cat# 115-225-146; RRID: AB_2307343) were used to detect *Gryllus* Tdc2 and octopamine, respectively. After five 30-min rinses in PBS-Tx, brains were dehydrated through 70%–100% ethanol and cleared with methyl salicylate.

Fluorescent images were captured using the Olympus FLUOVIEW FV1000 confocal laser scanning microscope (Olympus) or the Zeiss LSM 7 DUO laser scanning microscope (Zeiss). Microscopy parameters were adjusted to the brains of naïve crickets to prevent saturated pixels in the final images. For each brain, we captured a stack of optical sections using a 10× objective lens. Image processing was conducted using Fiji software (Schindelin et al., 2012; Schneider et al., 2012; RRID: SCR_002285). To visualize intensity of reporter protein expression, the EYFP immunoreactivity was displayed with “Red Hot” or “Magenta Hot” look-up tables.

### Behavioral experiments

Adult male crickets 1 week after the imaginal molt were used for behavioral experiments. Each cricket was individually isolated in a 100-ml beaker (ø4.5 cm) for 3 days without food and water before behavioral experiment.

#### Gustatory stimulation

Each cricket was fed a drop of 0.5 M sucrose solution (5 µl) three times with 5-min interval. 1 h after feeding, crickets were collected for RT-qPCR expression analysis. 6 h after feeding, crickets were collected for immunohistochemistry.

#### Agonistic behavior

A pair of weight-matched crickets (weight range: 0.6–0.8 g) were introduced into a round glass arena (ø12 cm) for agonistic interaction. We observed their behavior for 5 min, and winner and loser were determined according to following criteria: the winner sings an aggressive song and chases the loser, while the loser flees from the winner. In some cases, aggressive behavior did not escalate and dominance hierarchy was not established in 5 min. After 5-min interaction, each cricket was re-isolated in a 100-ml beaker. 1 h after the start of interaction, crickets were collected for RT-qPCR expression analysis.

### Data analysis

All statistical analyses were conducted using GraphPad Prism version 6.0 for Mac (GraphPad Software; RRID: SCR_002798). Box plots indicate 25th to 75th percentile ranges and central values, and ‘+’ indicates mean. Error bars indicate 5th to 95th percentile ranges. Asterisks donate statistical significance (****, *p <* 0.0001; ***, *p <* 0.001; **, *p <* 0.01; *, *p <* 0.05). Details on statistical analyses are described in Table 3.

**Table 3.** Experimental conditions and statistics

Figure	Experimental conditions	Statistical test
1*B–G*	Adult male crickets 1 week after the imaginal molt were isolated for 3 days. Crickets were injected with 20 mM cycloheximide. 1 h later, 5 mM PTX or vehicle were injected to the crickets. 30 min later, brains were dissected for RNA extraction (n = 8 in each group).	*Gryllus fra* total vehicle vs. *Gryllus fra* total PTX, *U* = 23; *Gryllus fra*-A vehicle vs. *Gryllus fra*-A PTX, *U* = 24, *Gryllus fra*-B vehicle vs. *Gryllus fra*-B PTX, *U* = 15; *Gryllus jra* vehicle vs. *Gryllus jra* PTX, *U* = 32; *Gryllus egr-B* vehicle vs. *Gryllus egr-B* PTX, *U* = 11; *Gryllus hr38* vehicle vs. *Gryllus hr38* PTX, *U* = 29, Mann-Whitney U test.
2*A*, *B*	Adult male crickets 1 week after the imaginal molt were isolated for 3 days. Crickets were injected with (A) 20 mM cycloheximide or (B) saline. 1 h later, 5 mM PTX was injected to the crickets. Brains were dissected for RNA extraction before PTX injection (0 min), or 15, 30, 45, 60, 90, or 120 min after PTX injection (n = 8 in each group).	Effect of pre-treatment: *F*(1,98) = 0.9604, *p* = 0.9004; Effect of time: *F*(6,98) = 9.034, *p* < 0.0001; interaction: *F*(6,98) = 0.3634, *p* = 0.9004, Two-way ANOVA, Dunnett’s post-hoc test.
2*C*	Same as in Fig. 2*A*.	*H* = 29.21, *p* < 0.0001, Kruskal-Wallis test, Dunn’s post-hoc test.
2*D*	Adult male crickets 1 week after the imaginal molt were isolated for 3 days. Cricket were fed with 0.5 M sucrose solution 3 times with 5 min intervals. Brains were dissected for RNA extraction before feeding (naïve), or 1 h after feeding (n = 8 in each group).	*t* = 3.051, *df* = 8.491, Welch’s unpaired *t*-test.
2*E*	Adult male crickets 1 week after the imaginal molt were isolated for 3 days. Weight-matched crickets were introduced into an arena to interact for 5 min, then re-isolated. Brains were dissected for RNA extraction before interaction (naïve), or 1 h after start of interaction (n = 8 in each group).	*H* = 12.62, *p* < 0.01, Kruskal-Wallis test, Dunn’s post-hoc test.
5*D*	Adult male crickets 1 week after the imaginal molt were isolated for 3 days. Then, brains were dissected for RNA extraction (n = 8 in each group).	*t* = 15.95 *df* = 7.094, Welch’s unpaired *t*-test.
5*E*	Adult male crickets 1 week after the imaginal molt were isolated for 3 days. 5 mM PTX was injected to the crickets. Brains were dissected for RNA extraction before PTX injection (0 min), or 15, 30, 45, 60, 90, or 120 min after PTX injection (n = 8 in each group).	*H* = 25.87, *p* < 0.001, Kruskal-Wallis test, Dunn’s post-hoc test.
5*F*	Same as in Fig. 5*C*.	*H* = 39.42, *p* < 0.0001, Kruskal-Wallis test, Dunn’s post-hoc test.
5*G*	Pooled expression data of *EYFPnls:PEST* and *Gryllus egr-B* in the brain within 120 min after PTX injection (including data presented in Fig. 5*C*, *D*), 60 min after vehicle injection, and naïve crickets were analyzed (n = 72, 8, and 8, respectively).	*r* = 0.8269, *p* < 0.0001, Pearson’s correlation analysis.
2-1*A*, *E*, *I*	Same as in Fig. 2*A*.	*Gryllus fra-A*, *F*(6,49) = 3.529, *p* < 0.001; *Gryllus fra-B, F*(6,49) = 3.327, *p* < 0.01; *Gryllus jra*, *F*(6,49) = 5.305, *p* < 0.001, One-way ANOVA, Dunnett’s post-hoc test.
2-1*B*, *F*, *J*	Pooled expression data of *Gryllus fra-A, fra-B, jra* and *egr-B* in the brain of cycloheximide pretreated crickets within 120 min after PTX injection (including data presented in Figs. 2-1*A*, *E*, and *I* and naïve crickets were analyzed (n = 48 and 8, respectively).	*Gryllus fra-A* vs. *egr-B, r* = 0.7856, *p* < 0.0001; *Gryllus fra-B* vs. *egr-B, r =* 0.5250, *p* < 0.0001*; Gryllus jra* vs. *egr-B*, *r* = 0.6222, *p* < 0.0001, Pearson’s correlation analysis.
2-1*C*, *G*, *K*	Same as in Fig. 2*D*.	*Gryllus fra-A*, *U* = 13; *Gryllus fra-B, U* = 7; *Gryllus jra*, *U* = 22, Mann-Whitney U test.
2-1*D*, *H*, *L*	Same as in Fig. 2*E*.	*Gryllus fra-A*, *H* = 10.24, *p* < 0.05; *Gryllus fra-B, H* = 13.09, *p* < 0.01; *Gryllus jra*, *H* = 2.284, *p =* 0.5156, Kruskal-Wallis test, Dunn’s post-hoc test.
1-5*B*, *C*	Adult male crickets 1 week after the imaginal molt were isolated for 3 days. Crickets were injected with (B) 20 mM cycloheximide or (C) saline. 1 h later, 5 mM PTX was injected to the crickets. Brains were dissected for RNA extraction before PTX injection (0 min), or 15, 30, 45, 60, 90, or 120 min after PTX injection (n = 8 in each group).	Effect of pre-treatment: *F*(1,98) = 2.060, *p* = 0.1544; Effect of time: *F*(6,98) = 14.57, *p* < 0.0001; interaction: *F*(6,98) = 0.7991, *p* = 0.5729, Two-way ANOVA, Šidák’s post-hoc test.
1-5*D*	Same as in Fig. 2*A, B*.	Effect of pre-treatment: *F*(1,98) = 0.5631, *p* = 0.4548; Effect of time: *F*(6,98) = 16.11, *p* < 0.0001; interaction: *F*(6,98) = 1.733, *p* = 0.1212, Two-way ANOVA, Šidák’s post-hoc test.
1-5*E*	Pooled expression data of *Gryllus hr38* and *Gryllus egr-B* in the brain of saline and cycloheximide pretreated crickets within 120 min after PTX injection (including data presented in Figs. 2*A*, *B* and Figs. 1-5*B*, *C*), and naïve crickets were analyzed (n = 48, 48, and 8, respectively).	Saline pretreated crickets: *r* = 0.8498, *p* < 0.0001; cycloheximide pretreated crickets: *r* = 0.8446; *p* < 0.0001; all data included: *r* = 0.8498, *p* < 0.0001, Pearson’s correlation analysis
1-5*F*	Same as in Fig. 2*D*.	*t* = 2.501, *df* = 13.18, Welch’s unpaired *t*-test.
1-5*G*	Same as in Fig. 2*E*.	*H* = 17.61, *p* < 0.001, Kruskal-Wallis test, Dunn’s post-hoc test.
2-2	Adult male crickets 1 week after the imaginal molt were isolated for 3 days. Crickets were received injection of PTX, forskolin, TPA, SNAP, anisomycin, A23187, or vehicle. Brains were dissected for RNA extraction 60 min after injection (PTX, n = 8; the other treatments, n = 16).	*Gryllus fra-A*, *H* = 62.94, *p* < 0.0001; *Gryllus fra-B, H* = 40.22, *p* < 0.0001; *Gryllus jra*, *H* = 56.30, *p* < 0.0001; *Gryllus egr-B, H* = 39.41, *p* < 0.0001; *Gryllus hr38, H* = 67.07, *p* < 0.0001, Kruskal-Wallis test, Dunn’s post-hoc test.

## Results

### *Gryllus egr* homolog is expressed as a neuronal IEG in the cricket brain

Although the activity-regulated genes or neuronal IEGs have not been identified in any hemimetabolous insects including the cricket, recent genome-wide analyses of activity-regulated genes in vertebrates (Nedivi et al., 1993; Matsuo et al., 2000; Wada et al., 2006; Spiegel et al., 2014) and several insect species (Guan et al., 2005; Chen et al., 2016; Ugajin et al., 2018) highlighted evolutionarily conserved transcription factor IEGs. On the basis of previous reports, we selected four transcription factor genes as our candidates for activity-regulated genes in the cricket as follows: *fos-related antigen* (*fra*; *c-fos* homolog), *jun-related antigen* (*jra*; *c-jun* homolog), *early growth response* (*egr*; *egr-1/zif268/NGFI-A* homolog), and *hormone receptor 38* (*hr38*; *NGFI-B/nur77/NR4A1* homolog).

First, we cloned cDNAs encoding four candidate activity-regulated genes from the cricket brain cDNA library. We isolated cDNAs encoding two transcript variants of *Gryllus fra* (*fra-A* and *-B* isoforms; LC215243 and LC215244, respectively), *Gryllus jra* (LC215245), *Gryllus egr* (LC215246), and *Gryllus hr38* (LC341255; see Figs. 1-2, 1-3, 1-4, and 1-5). In *Drosophila*, the *stripe* gene (*Drosophila egr* homolog) has two alternative promoters to express alternative transcripts (*stripe-A* and *-B;* Frommer et al., 1996). The use of alternative promoters was also known in the honeybee. In the honeybee brain, variant I and II of *AmEgr* (equivalent to *egr*/*stripe*-A of other insects) are transiently expressed during metamorphosis, whereas the other isoform, variant III (equivalent to *egr*/*stripe*-B), is induced in an activity-dependent manner (Ugajin et al., 2016). Although we did not obtain cDNA clones of *egr-A* in the cricket, we concluded that the *egr* gene expressed in the cricket brain is a homolog of *egr-B* found in other insects based on the similar structural (Fig. 1-4*C* ) and expression characteristics.

Next, we examined whether the expressions of the candidate activity-regulated genes are rapidly induced in the cricket brain in an activity-dependent manner. In this study, the γ-aminobutyric acid A (GABA_A_) receptor blocker picrotoxin (PTX) was used to cause neuronal hyperexcitability (Saffen et al., 1988; Ugajin et al., 2013). In the cricket, injection of 3 µl of 5 mM PTX caused immediate locomotor hyperactivity and, in turn, seizure-like behavior (e.g., abdominal contraction and shaking of appendages). The effect of PTX persisted at least 30 min, and most crickets recovered from the seizure within 90 min after injection. In addition, to clarify whether the candidate genes are induced as neuronal IEGs, 20 mM cycloheximide was injected 1 h before PTX injection to block *de novo* protein synthesis (Matsumoto et al., 2003). Quantitative RT-PCR analysis revealed that, 30 min after PTX injection, the expression levels of *Gryllus fra-B* and *egr-B* were significantly elevated in the brains of PTX-injected crickets (∼1.5-fold up-regulation) versus those of vehicle-injected crickets (Fig. 1). These data demonstrated that, in the cricket brain, *Gryllus fra-B* and *egr-B* were rapidly induced as neuronal IEGs in response to neuronal hyperexcitability.

Next, we examined expression time course of activity-regulated genes in the cricket brain after PTX injection, especially focused on *Gryllus egr-B* (Fig. 2). In the brains of cycloheximide pretreated crickets, the expression of *Gryllus egr-B* reached a maximum 60 min after PTX injection [∼4.5-fold up-regulation relative to both the naïve animals and the control (0 min after PTX injection)] and decreased to near baseline level by 120 min after PTX injection (Fig. 2*A*). To ensure that the activity-dependent transcriptional activation of *Gryllus egr-B* is composed only of an immediate-early component, we compared the expression kinetics of *Gryllus egr-B* after PTX injection with or without the administration of cycloheximide (Fig. 2*A*, *B*). The expression kinetics of *Gryllus egr-B* was not affected by blockade of *de novo* protein synthesis (two-way ANOVA, effect of pretreatment: *F*(1,98) = 0.9604, *p =* 0.3295; effect of time: *F*(6,98) = 9.034, *p <* 0.0001; interaction: *F*(6,98) = 0.3634, *p =* 0.9004), indicating *Gryllus egr-B* was induced as an IEG in the cricket brain after PTX injection. Next, we examined when PTX-induced *Gryllus egr-B* is actively transcribed by examining the expression time course of *Gryllus egr-B* premature mRNA (pre-mRNA). In the brains of cycloheximide-pretreated crickets, the expression of *Gryllus egr-B* pre-mRNA significantly increased within 15 min after PTX injection [∼7-fold up-regulation relative to both the naïve animals and the control (0 min after PTX injection)], was sustained at a high level for >1 h, and then decreased to near baseline level by 120 min after injection (Fig. 2*C*). These data indicate that, after PTX injection, transcription of *Gryllus egr-B* is rapidly induced and sustained at a high level while its corresponding mRNA is elevated in the brain. Finally, we tested behaviorally evoked expression of *Gryllus egr-B*. The expression of *Gryllus egr-B* in the brain was significantly increased 1 h after feeding of sucrose solution and agonistic interaction (Fig. 2*D*, *E*). These data indicated that *Gryllus egr-B* is induced as a neuronal IEG in response to strong, sustained, widespread neuronal hyperexcitability caused by PTX, as well as behaviorally evoked neuronal activation in the cricket brain.

10.1523/ENEURO.0061-18.2018.f2-1 Figure 2-1 Expression characteristics of bZip transcription factor genes in the cricket brain. Expression characteristics of (***A–D***) *Gryllus fra-A* (***E–H***) *fra-B*, and (***I–K***) *jra*. ***A***, ***E***, ***I***, Expression time course of *Gryllus fra-A*, *fra-B*, and *jra* after PTX-injection in the brains of cycloheximide-pretreated crickets. The expression levels were normalized to the mean of those of the naïve animals (baseline expression level). The expressions of *Gryllus fra-A* and *fra-B* reached a maximum 60 min after PTX injection (*fra-A*, ∼3.5-fold and ∼2.5-fold up-regulation relative to the naïve animals and to the control (0 min after PTX injection), respectively; *fra-B*, ∼2-fold up-regulation relative to both the naïve animals and to the control (0 min after PTX injection), whereas that of *Gryllus jra* reached a maximum 45 min after PTX injection (∼6-fold and ∼2-fold up-regulation relative to the naïve animals and to the control, 0 min after PTX injection, respectively). ***B***, ***F***, ***J***, Correlation plot between the expression levels of *Gryllus fra-A*, *fra-B*, and *jra* with *Gryllus egr-B* in the brains of cycloheximide-pretreated crickets. The data from the cycloheximide and PTX-injected crickets (n = 48; yellow circles) and naïve crickets (n = 8; black circles) were plotted. Behaviorally evoked expression of *Gryllus fra-A*, *fra-B*, and *jra* in the brain of crickets 1 h after (***C***, ***G***, ***K***) sucrose feeding and (***D***, ***H***, ***L***) agonistic interaction. The expression levels were normalized to the mean of those of the naïve animals (baseline expression level). RT-qPCR analyses were performed on eight biological replicates. Box plots indicate the 25th to 75th percentile ranges and central values. Error bars indicate the 5th to 95th percentile ranges. The “+” denotes the mean. Asterisks donate statistical significance to the control (0 min after PTX injection; ***A***, ***E***, ***I***) or to the naïve animals (***C***, ***D***, ***G***, ***H***, ***K***, ***L***; *, *p* < 0.05; **, *p* < 0.01). See Table 3 for the details of statistical analysis. Download Figure 2-1, EPS file.

10.1523/ENEURO.0061-18.2018.f2-2 Figure 2-2 Pharmacologically induced expression of neuronal IEGs in the cricket brain. Pharmacologically induced expression of (***A***) *Gryllus fra-A*, (***B***) *fra-B*, (***C***) *jra,* (***D***) *egr-B*, and (***E***) *hr38* in the brain of crickets. Following activators were used to stimulate intracellular signaling pathways: forskolin, an activator for adenylyl cyclases; 12-*O*-tetradecanoylphorbol-13-acetate (TPA), an activator for protein kinase C; anisomycin, an activator for c-Jun N-terminal kinases; S-nitroso-*N*-acetylpenicillamine (SNAP), a nitric oxide donor; and a calcium-selective ionophore A23187. PTX was used as a positive control. The expression levels were normalized to the mean of those of the naïve animals (baseline expression level). Injection of 200 µM TPA resulted in significant increases in the expression levels of all neuronal IGEs (***A–D***). Injection of 200 µM forskolin and 200 µM A23187 resulted in significant increases in the expression levels of *fra-A* and *jra*, respectively. No obvious change was observed with any drug other than TPA in the expression levels of *Gryllus fra-B* and *egr-B* (***B*** and ***D***). The expression level of *Gryllus hr38* was drastically affected by stimulation of intracellular signaling pathways (***E***). RT-qPCR analyses were performed on 8 or 16 biological replicates. Box plots indicate the 25th to 75th percentile ranges and central values. Error bars indicate the 5th to 95th percentile ranges. The “+” denotes the mean. Letters above the plots (a and b) indicate statistical significance (*p* < 0.05) to the naïve control and vehicle control, respectively. See Table 3 for the details of statistical analysis. Download Figure 2-2, EPS file.

Although we could not detect significant increase of *Gryllus fra-A*, *jra*, and *hr38* 30 min after PTX injection in the initial expression analysis (Figs. 1*A*, *C*, *D*), these genes are slowly up-regulated in the brain under the influence of cycloheximide after PTX injection (Figs. 1-5 and 2-1). The expression of *Gryllus fra-A*, *fra-B*, and *hr38* were elevated 1 h after feeding of sucrose solution, whereas those of *Gryllus fra-B* and *hr38* were elevated 1 h after agonistic interaction. These data indicate that all candidate activity-regulated genes were expressed as neuronal IEGs in the brain with gene-specific expression kinetics and regulation mechanisms.

### *Cis*-regulatory elements for stimulus-induced gene expression are enriched in the upstream genomic regions of insect/crustacean *egr-B* homologs

To construct an IEG promoter-driven reporter system, we determined the nucleotide sequences of the gene regulatory region of *Gryllus egr-B.* By using RNA ligase-mediated 5′ RACE and inverse PCR, we determined the transcription start site (TSS) and obtained a ∼1.6-kbp upstream genomic fragment of *Gryllus egr-B*. Sequence comparison of the upstream regions of *egr-B* homologs revealed that the core promoter region (∼200 bases flanking the TSS) of *Gryllus egr-B* showed a high level of sequence similarity with the corresponding part of most of *egr-B* homologs of basal insects (hemimetabolous insects + Coleoptera [*Tribolium castaneum*]) and the crustacean *Daphnia pulex* (Fig. 3*A*; See Table 2). The core promoter region of basal insect/crustacean *egr-B* contained *cis*-regulatory elements regulating stimulus-dependent gene expression (two CREs and SRE), as well as sequence elements for core promoter function such as a CCAAT-box, initiator element (Inr; consensus: YCATTC), and a downstream promoter element (DPE; consensus: AGTYY; see Fig. 3*A*, *B*). Additionally, we found that two more sequence motifs, a GAGA motif and a GC-rich motif, which are structurally related to the promoter elements associated with RNA polymerase II (Pol II) stalling in *Drosophila* (Hendrix et al., 2008), are conserved in the upstream sequences of *egr-B* homologs of most hemimetabolous insects (Fig. 3*A*, *B*). The GAGA motif (consensus: GRGAGGGRVGGAGAGS) is conserved in the upstream sequences of polyneopteran *egr-B* homologs positioned at ∼80 bp upstream to the TSS of *Gryllus egr-B*, but lacking in those of *egr-B* homolog of the other taxa (i.e., Paraneoptera, Coleoptera, and crustacea). The GC-rich motif (consensus: GCGCSSSGGCGCGC) is conserved in the upstream sequences of *egr-B* homolog of basal insects positioned at ∼30 bp downstream to the TSS of *Gryllus egr-B*.

10.1523/ENEURO.0061-18.2018.f3-1 Figure 3-1 Sequence comparison of the DNA-binding domain of stimulus-regulated transcription factors. The amino acid sequence of the DNA-binding domain of stimulus-regulated transcription factors of mouse and several insect species were aligned. (***A***) Fos family, (***B***) Jun family, (***C***) ATF2, (***D***) ATF3, (***E***) ATF4/5, (***F***) ATF6, (***G***) large Maf family, (***H***) small Maf family, (***I***) CREB1 family, (***J***) insect CREB-B family, (***K***) C/EBPs except for C/EBPγ and C/EBPζ, (***L***) C/EBPγ, (***M***) Egr family, (***N***) NFAT family, (***O***) MEF2 family, and (***P***) SRF. The residues for protein–DNA interaction were highly conserved in Jun family proteins, large/small Maf family proteins, CREB-like proteins, C/EBPs except for C/EBPγ, MEF2, and SRF (see ***A***, ***G–K***, and ***O–P***). One or few substations were detected in Fos family proteins, Egr and NFAT homologs (see ***A***, ***M***, and ***N***). Extensive substitutions were found in the DBD of ATF4/5, ATF6, and C/EBPγ (see ***B*** and ***F***). A lineage-specific occurrence of substitutions in the DBD of insect ATF2 homologs (see ***C***). That is, extensive substitutions were detected in ATF2 homologs of *Drosophila*, *Apis*, and *Tribolium* (“advanced” holometabolous insects), whereas the amino acid sequence of the ATF2 DBD is conserved between mouse and *Zootermopsis* (a “basal” hemimetabolous insect). The conserved residues are marked with asterisks above the alignments. Residues important for nucleotide binding were indicated by black circles under the alignments. Red circles indicate the residues important for nucleotide binding where amino acid substitutions were found in most insect homologs. Residues important for protein–protein interaction (i.e. dimerization) were indicated by white circles under the alignments. The amino acid residues are represented in the default color scheme of ClustalX. GenBank IDs of proteins used for sequence comparison are listed in Table 3-1. Download Figure 3-1, EPS file.

10.1523/ENEURO.0061-18.2018.t3-1 Table 3-1 List of stimulus-regulated transcription factors (TFs) in mammals and their homologs in insects. Download Table 3-1, DOC file.

Next, we expanded our analysis to the distal promoter region by predicting potential *cis*-regulatory motifs (Fig. 3*C*, see also Fig. 3-1). Generally, the upstream regions of insect/crustacean *egr-B* homologs were highly enriched with potential binding sites for transcription factors activated by various intracellular signaling pathways (e.g., AP-1, C/EBP, CREB, SRF, and NF-AT), which are known to regulate activity-dependent gene expression in vertebrates (Sealy et al., 1997; Hogan et al., 2003; Benito et al., 2011; Nonaka et al., 2014). In addition, phylogenetic footprinting analysis of the upstream region of *egr-B* homologs of polyneopteran insects revealed two conserved *cis*-regulatory modules (CRMs; CRM_-800_ and CRM_-400_ in Fig. 4*A*), which consist of proximal, conserved potential binding sites for stimulus-regulated transcription factors (Fig. 4*B*). These data indicate that the ∼1.6-kbp upstream genomic fragment of *Gryllus egr-B* contains *cis*-regulatory elements for stimulus-regulated transcription factors, some of which constitute the core promoter and CRMs conserved across insect/arthropod species.

### Construction of the IEG promoter-driven transgenic reporter system

An IEG promoter-driven reporter system was constructed using the upstream genomic fragment of *Gryllus egr-B* (Fig. 5*A*, *B*). The IEG reporter cassette consisted of a nuclear-targeted destabilized EYFP (*EYFPnls:PEST*; Li et al., 1998) driven by a ∼2.2-kb genomic DNA fragment containing the ∼1.6-kbp upstream sequence and 5′ UTR of *Gryllus egr-B*. Two repeats of a synthetic AU-rich element were inserted between the coding sequence of *EYFPnls:PEST* and the SV40 3′ UTR to shorten mRNA half-life (Pham et al., 2008). The IEG reporter cassette along with a *3xP3-mCherry* expression cassette was integrated into the cricket genome using the *piggyBac* transposon. Successful transgenic crickets were selected according to mCherry fluorescence in the compound eyes, and reporter expression in the brain was checked by EYFP immunoreactivity after PTX treatment. In the *Drosophila* brain, Masuyama et al. (2012) reported that the inducible expression of EGFP protein under the control of activity-dependent synthetic transcription factor, CaLexA, became detectable ∼4 h after stimulation. In the present study, we collected brains of the IEG reporter line 6 h after PTX injection to detect activity-dependent expression of reporter protein. Three of 37 mCherry-expressing transgenic lines showed EYFP immunoreactivity in the brain after PTX injection. One transgenic line with a low background EYFP expression (line #19; Fig. 5*C*) was selected, and a homozygous transgenic line was established as an “IEG reporter line” for subsequent analyses. The IEG reporter line develops normally, and we did not observe any behavioral abnormality.

We tested whether the expression of IEG reporter mimics innate IEG expression in the brains of the IEG reporter line. First, we compared baseline expression levels of the reporter gene (*EYFPnls:PEST*) and *Gryllus egr-B*. Quantitative RT-PCR expression analysis revealed that, in the brains of naïve animals, *EYFPnls:PEST* showed a higher baseline expression level than *Gryllus egr-B* (Fig. 5*D*). In addition, Time course analysis of PTX-induced expression of *EYFPnls:PEST* and *Gryllus egr-B* revealed differences in the expression kinetics of the genes after PTX injection. The expression of *EYFPnls:PEST* reached a maximum 60–90 min after PTX injection (∼1.8-fold up-regulation relative to the naïve animals), and slowly decreased to near baseline by 120 min after injection (Fig. 5*E*). On the other hand, the expression of *Gryllus egr-B* reached a maximum 90 min after PTX injection (∼6.5-fold up-regulation relative to the naïve animals) and decreased to the baseline level by 120 min after PTX injection (Fig. 5*E*). Correlation analysis revealed that the expression level of the *EYFPnls:PEST* was strongly correlated with that of *Gryllus egr-B* in the brain of the IEG reporter line (Pearson’s *r =* 0.8269, *p <* 0.0001; Fig. 5*F*). With these data, we concluded that the neuronal activity-driven expression of the reporter gene mimics that of *Gryllus egr-B* in the brain of the IEG reporter line.

### IEG promoter-driven reporter system can be used for whole-brain activity mapping

**Figure 6.**
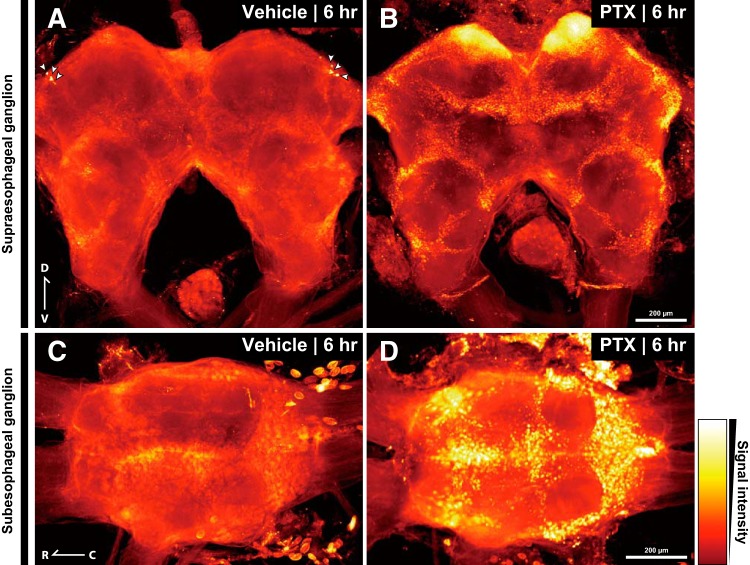
PTX-induced reporter protein expression in the brain of the IEG reporter line. Distribution of the reporter protein (EYFPnls:PEST) in the brain of the IEG reporter line was examined by whole-mount fluorescent immunohistochemistry. ***A***, ***B***, Frontal views of the supraesophageal ganglion stained with anti-GFP antibody. ***A***, EYFP immunoreactivity was only observed in the cells is indicated by white arrowheads 6 h after vehicle injection. ***B***, EYFP immunoreactivity was observed throughout the ganglion 6 h after PTX injection. ***C***, ***D***, Ventral views of the subesophageal ganglion stained with anti-GFP antibody. ***C***, EYFP immunoreactivity was not observed 6 h after vehicle injection. ***D***, EYFP immunoreactivity was observed throughout the ganglion 6 h after PTX injection. Dorsoventral (D-V) or rostrocaudal (R-C) axes were indicated. Scale bars represent 200 µm. See Movies 1 and 2 for the full stack of optical sections of the supraesophageal ganglia shown in ***A*** and ***B***.

First, we asked whether our reporter system can be used for whole-brain activity mapping. The brains of the IEG reporter line were collected 6 h after PTX or vehicle injection, and the distribution of the reporter protein (EYFPnls:PEST) was examined by whole-mount fluorescent immunohistochemistry (Fig. 6). In the brain of vehicle-injected animals (*n* = 7), intense EYFP immunoreactivity was only detected in cell clusters located in the lateral parts of the supraesophageal ganglion (white arrowheads in Fig. 6*A*, *C*). The distribution of the EYFPnls:PEST protein in the brain of naïve animals was similar to that in the vehicle-injected animals (data not shown). Six hours after PTX injection, EYFP immunoreactivity was detected throughout the ganglia (Fig. 6*B*, *D*; *n* = 6) and was restricted to the nucleus. In addition, we did not observe EYFP immunoreactivity in the marginal glia (the cells located at the margins of the neuropil) or the epithelial glia after PTX injection, suggesting that the reporter protein is only induced in the neurons (Movies 1 and 2). To examine the expression time course of the reporter protein, we conducted Western blot analysis using several anti-GFP antibodies. However, we could not detect bands corresponding to EYFPnls:PEST before and after PTX injection. We speculated that restricted localization of the reporter protein in the nuclei might facilitate detection of the reporter protein in immunohistochemistry.

Movie 1. EYFP immunoreactivity in the optical sections of the supraesophageal ganglion of IEG reporter line 6 h after vehicle injection. 10.1523/ENEURO.0061-18.2018.video.1

Movie 2. EYFP immunoreactivity in the optical sections of the supraesophageal ganglion of IEG reporter line 6 h after PTX injection stained with anti-GFP antibody. 10.1523/ENEURO.0061-18.2018.video.2

Next, we tested whether our reporting system can label behaviorally evoked neural activation under the physiological conditions. As our RT-qPCR analysis revealed that feeding of sucrose solution induced expression of neuronal IEGs in the cricket brain, we examined the expression of the reporter protein induced by feeding of sucrose solution in the brain of the IEG reporter line. In the insect brain, the octopaminergic/tyraminergic neurons are activated by feeding of sucrose solution or presentation of “reward” in the context of learning and memory studies. In the honeybee, an identified octopaminergic neuron, VUMmx1, in the subesophageal ganglion responds to sucrose solution applied to the antenna and proboscis (Hammer, 1993). In *Drosophila*, the octopaminergic/tyraminergic neurons in the subesophageal ganglion control larval appetite (Zhang et al., 2013) and mediate reward signaling in both larvae and adult flies (Burke et al., 2012; Selcho et al., 2014). Furthermore, in the cricket, the pharmacological blockade of α-adrenergic-like octopamine receptor impaired appetitive learning, indicating that the octopaminergic system is involved in reward signaling (Unoki et al., 2005, 2006; Matsumoto et al., 2015). These allow us to hypothesize that the feeding of sucrose solution would activate the octopaminergic/tyraminergic system in the subesophageal ganglion of the cricket. According to this idea, we asked whether the feeding of sucrose solution induces the reporter protein expression in the octopaminergic/tyraminergic neurons in the subesophageal ganglion, especially focused on the dorsal unpaired median (DUM) neurons (Pflüger and Stevenson, 2005).

**Figure 7.**
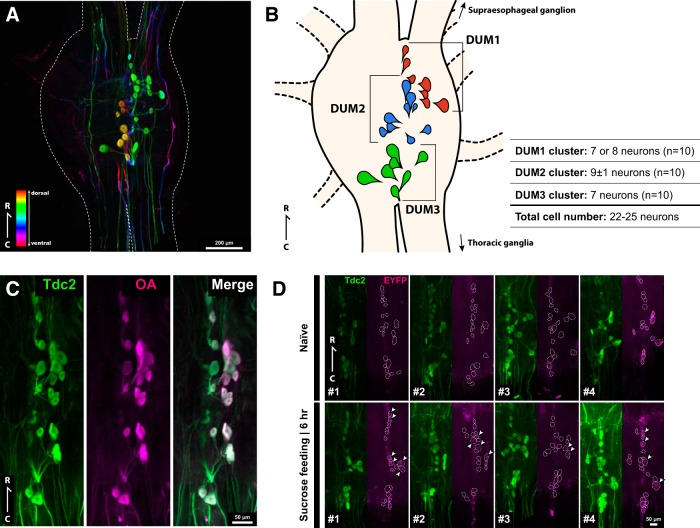
Sucrose feeding-evoked reporter protein expression in the DUM neurons of the IEG reporter line. ***A***, Dorsal view of the subesophageal ganglion of the Hokudai WT strain stained with anti-*Gryllus* Tdc2 antibody. The outline of the ganglion is surrounded by the white dotted line. The depth of the cells is color coded as indicated in the inset. Rostrocaudal (R-C) axis was indicated. Scale bar represents 200 µm. See Fig. 7-1 for the octopamine biosynthesis pathway and the structures of the Tdc proteins in insects. See Fig. 7-2 for the frontal view of the supraesophageal ganglion and the ventral view of the subesophageal ganglion stained with anti-*Gryllus* Tdc2 antibody. ***B***, Schematic drawing of the positions and numbers of the cell bodies of three DUM clusters (DUM1, DUM2, DUM3) on the dorsal side of the subesophageal ganglion. ***C***, Double fluorescent immunostaining confirmed that the DUM neurons contain octopamine. The DUM neurons were stained with the anti-*Gryllus* Tdc2 antibody (green) and anti-octopamine antibody (magenta). Scale bar represents 50 µm. ***D***, Distribution of the reporter protein (EYFPnls:PEST) in the DUM neurons of the IEG reporter line before and 6 h after feeding of sucrose solution (*n* = 4 each). The DUM neurons were stained with the anti-*Gryllus* Tdc2 antibody (green) and anti-GFP antibody (magenta). The cell bodies of *Gryllus* Tdc2 immunoreactive DUM neurons are surrounded by the white dotted line. The DUM neurons with nuclear EYFP immunoreactivity are indicated by white arrowheads. Scale bar represents 50 µm.

To label the octopaminergic/tyraminergic neurons in the cricket brain, we first generated anti-*Gryllus* tyrosine decarboxylase 2 (Tdc2) antibody. In *Drosophila*, Tdc2 is responsible for the production of tyramine (a precursor of octopamine) in the nervous tissues (Cole et al., 2005; see Fig. 7-1), and the *tdc2-Gal4* driver is used for targeted gene expression in the octopaminergic/tyraminergic neurons in *Drosophila*. Our anti-*Gryllus* Tdc2 antibody successfully immunolabeled the octopaminergic/tyraminergic DUM neurons in the subesophageal ganglion of the Hokudai WT strain (Fig. 7*A*, *B*; see also Fig. 7-2), which is confirmed by double immunostaining with the anti-octopamine antibody (Fig. 7*C*). Then, we examined whether reporter protein expression becomes detectable in the nuclei of the DUM neurons in response to the feeding of sucrose solution. In the subesophageal ganglia of the naïve IEG reporter line, the DUM neurons did not show nuclear-localized staining of EYFP immunoreactivity (*n* = 4; Fig. 7*D*, upper columns). As we expected, 6 h after feeding of sucrose solution, the nuclear-localized reporter protein expression was detected in several neurons in the DUM1 and DUM2 clusters (*n* = 4; Fig. 7*D*, lower columns). These data demonstrate that our IEG promoter-driven reporter system can visualize neuronal activation caused by PTX-induced neuronal hyperexcitability, as well as behaviorally evoked neuronal activation.

10.1523/ENEURO.0061-18.2018.f7-1 Figure 7-1 Molecular cloning of *Gryllus Tdc* genes. ***A***, Octopamine biosynthesis pathway in the insect brain. Tdc catalyzes the first step of octopamine biosynthesis by converting L-tyrosine into tyramine. ***B***, ***C***, Insect Tdc proteins contain subtype-specific C-terminal extensions. Comparison of the amino acid sequences of the C-terminal extensions of (***B***) *Gryllus* Tdc1 and (***C***) Tdc2 with their corresponding parts of Tdc homologs in other insects. The conserved residues are marked with asterisks above the alignments. The amino acid residues are represented in the default color scheme of ClustalX. Positions of the C-terminal portion of the catalytic domains were indicated by bars under the alignment. GenBank IDs of proteins are following: *D. melanogaster* Tdc1, NP_610226; *D. melanogaster* Tdc2, NP_724489; *A. aegypti* Tdc1, XP_001656851; *A. aegypti* Tdc2, XP_001656857; *T. castaneum* Tdc1, XP_972728; *T. castaneum* Tdc2, XP_972688. Download Figure 7-1, EPS file.

10.1523/ENEURO.0061-18.2018.f7-2 Figure 7-2 Distribution of the Tdc2-expressing neurons in the cricket brain. ***A***, ***B***, Frontal (***A***) view of the supraesophageal ganglion and ventral (***B***) view of the subesophageal ganglion stained with anti-*Gryllus* Tdc2 antibody. The outlines of the ganglia are indicated by the white dotted lines. The depth of the cells is color coded as indicated in the inset. Dorsoventral (D-V) or rostrocaudal (R-C) axes were indicated. Scale bars represent 200 µm. Download Figure 7-2, EPS file.

## Discussion

In the present study, we established an IEG promoter-driven transgenic reporter system for whole-brain activity mapping in the cricket. In the cricket brain, the neuronal IEGs, *Gryllus fra-B*, *egr-B*, and *hr38*, were up-regulated within 1 h after PTX-induced neuronal hyperexcitability, and after feeding of sucrose solution and agonistic interaction. We found that the region ∼1.6 kbp upstream of *Gryllus egr-B* is sufficient to drive reporter gene expression in response to neuronal hyperexcitability. Inducible reporter protein expression was broadly detected in the brain of the IEG reporter line. Finally, we detected feeding-evoked neuronal activations in the cricket brain with our reporter system. Our study also revealed that the core promoter region of *egr-B* homologs show a high level of structural conservation in basal insects and crustaceans.

The *egr-1/zif268/NGFI-A* gene was first identified as an IEG induced by a variety of extracellular stimuli (e.g., serum, mitogens, and growth factors) in various cell types (Sukhatme et al., 1987; Milbrandt, 1987), and was later shown to be induced after depolarization in neurons (Sukhatme et al., 1988). Then, it became widely used as a marker for neuronal activation in various vertebrate species (Brennan et al., 1992, 1999; Mello and Clayton, 1994; Hoke et al., 2004; Burmeister and Fernald, 2005). In the cricket brain, *Gryllus egr-B* was rapidly induced as an IEG in response to neuronal activation. Neuronal activity–regulated expression of *egr* homologs was also reported in the brain of *Drosophila* (Guan et al., 2005; Chen et al., 2016) and the honeybee (Ugajin et al., 2013). These data support the idea that the *egr-1/zif268/NGFI-A* homologs are evolutionarily conserved neuronal IEGs between vertebrates and insects.

In the present study, using the promoter region of *Gryllus egr-B* and the nuclear-targeted destabilized EYFP as a reporter, we established a retroactive, whole-mount, single-cell-resolution activity mapping system to highlight behaviorally relevant neuronal circuits in the cricket brain. Transgenic reporter systems for neuronal activity mapping have been constructed in the mouse and *Drosophila* by using innate activity-dependent promoters (Smeyne et al., 1992; Wang et al., 2006; Reijmers et al., 2007; Barth, 2007; Kim et al., 2015), as well as synthetic promoters (Kawashima et al., 2013; Fujita et al., 2013; Sørensen et al., 2016) or a synthetic activity–regulated transcription factor (Masuyama et al., 2012). In rodents, the activity-dependent promoters were used to express the channelrhodopsin gene in the behaviorally relevant neurons to investigate the formation of memory engrams (Garner et al., 2012; Liu et al., 2012; Ramirez et al., 2013). In our future research, we will combine our IEG promoter-based gene expression system with optogenetic/chemogenetic tools or genetically encoded calcium indicators to uncover the key neural circuits involving cricket social behaviors. Moreover, recent advances in genetic engineering techniques (transposon-based germline transgenesis and CRISPR/Cas-based knock-in; O’Brochta and Atkinson, 1996; Atkinson et al., 2001; Kimura, 2001; Handler, 2002; Sun et al., 2017) allow us to introduce the IEG promoter-based reporter system into non-*Drosophila* insects classically used in insect neuroethology.

When neuronal IEG mRNAs are induced in the vertebrate nervous system, expression reaches its peak around 30 min after stimulation, and then rapidly returns to baseline by 60 min (Zangenehpour and Chaudhuri, 2002, Mello and Clayton, 1994, Burmeister et al., 2005). On the contrary, in the cricket brain, the expression levels of neuronal IEGs peaked more slowly (60–90 min after PTX injection; Figs. 2, 1-5, and 2-1). This is consistent with previous studies in other insect species (Kiya et al., 2007; Ugajin et al., 2012, 2016, 2018; Fujita et al., 2013). These data indicate that, in general, mRNAs of insect neuronal IEGs show slower expression kinetics than those in the vertebrate systems. The expression dynamics of inducible gene mRNA is determined depending on the rates of transcription and mRNA decay (Shalem et al., 2008). In the cricket brain, transcription of *Gryllus egr-B* was rapidly initiated on stimulation, and promoter activity was sustained at a high level for >1 h after stimulation. In the vertebrate system, neuronal IEGs are categorized into two subgroups: rapid and delayed IEGs (Saha and Dudek, 2013). The expression of delayed IEGs requires several time-consuming steps to recruit Pol II to the promoter for the initiation of transcription (e.g., signal transduction, posttranslational modifications of transcription factors, and chromatin remodeling), whereas transcription of rapid IEGs is initiated by stalled Pol II. In insects, Pol II stalling–dependent rapid transcription initiation was found in *Drosophila* heat shock response genes (Teves and Henikoff, 2013), where inducible gene expression becomes detectable within 5 min after stimulation (Vazquez et al., 1993). Rapid initiation of *Gryllus egr-B* transcription suggests that this gene is expressed as a rapid IEG in the cricket brain. This is also supported by the existence of promoter elements for Pol II stalling in the core promoter region of *Gryllus egr-B*, which is discussed in detail in the following section. The level of *Gryllus egr-B* mRNA is decreased when its promoter activity is down-regulated, indicating that *Gryllus egr-B* mRNA undergoes rapid degradation. Our expression analysis also revealed that another neuronal IEG, *Gryllus hr38*, has a slower induction kinetics with exceptionally long mRNA half-life when compared to the case of *Gryllus egr-B* (Fig. 1-5), suggesting that *Gryllus hr38* is expressed as a delayed IEG in the cricket brain.

As mentioned above, the core promoter region of *Gryllus egr-B* contained promoter elements associated with Pol II stalling, which are highly conserved in the upstream region of basal insects/crustacean *egr-B* homologs. Pol II stalling was first reported on *Drosophila* heat shock genes (Gilmour and Lis, 1986). Now, Pol II stalling is considered as a widespread mechanism of transcription regulation for rapid and precise control of gene expression in metazoans (Core and Lis, 2008; Nechaev et al., 2010; Levine, 2011). Genome-wide survey in *Drosophila* (Hendrix et al., 2008) revealed that ∼20% of stalled promoters contain an arrangement of core promoter elements, namely, GAGA motif, Inr, GC-rich motif, and/or DPE. Our promoter analysis revealed that all of the core promoter elements found in *Drosophila* stalled promoters are contained in the core promoter region of *Gryllus egr-B* and basal insect *egr-B* homologs. The GAGA motif is found in the *Drosophila hsp70* promoter (Shopland et al., 1995; Wilkins and Lis, 1997) and other stalled promoters (Lee et al., 2008; Hendrix et al., 2008). In *Drosophila*, a GAGA transcription factor, GAF, occupies the GAGA motif in the stalled promoter and recruits transcription coregulators, such as the nucleosome remodeling factor and the negative elongation factor, to the core promoter region (Fuda et al., 2015; Tsai et al., 2016). In *Drosophila*, the Inr and DPE are enriched by ∼2-fold in the stalled promoters relative to the core promoter region of constitutively expressed genes, respectively (Hendrix et al., 2008). These elements are highly conserved in the core promoter of basal insect *egr-B* homologs, as are some sequence homologies of *Drosophila* stalled promoters (Hendrix et al., 2008). In the present study, we found a GC-rich motif between the TSS and DPE of the core promoter region of basal insect *egr-B* homologs. In *Drosophila* embryo, ∼25% of stalled promoters contain a GC-rich region between +1 and +50, which could promote Pol II stalling by attenuating the movement of the Pol II complex (Hendrix et al., 2008). To our surprise, the putative core promoter region of *Daphnia egr-B* homolog shares the similar arrangement of *cis*-regulatory and core promoter elements found in the upstream sequence of basal insect *egr-B* homologs (Fig. 3*A*). This finding indicates that ancestral arthropods acquired a prototypic *egr-B* core promoter before the diversification of insects from crustaceans (∼500 million years ago; Misof et al., 2014). To our knowledge, the core promoter region of insect/crustacean *egr-B* homologs is the most ancestral structurally conserved activity-regulated promoter.

In addition to the structural analysis of the core promoter, in the present study we investigated the spatial distribution of *cis*-regulatory elements in the upstream region of insect *egr-B* homologs. In vertebrates, structural and functional analyses of activity-regulated promoters/enhancers revealed transcription factor binding sites essential for inducible gene expression (West et al., 2002; Benito and Barco, 2015). For example, Kawashima et al. (2009) identified a synaptic activity-responsive CRM at ∼7 kb upstream of the TSS of the mouse *Arc* gene, which consists of closely localized binding sites for CREB, MEF2, and SRF. The promoter region of the *c-fos* gene contains binding sites for SRF and CREB, which play crucial roles in activity-regulated gene expression (Ghosh et al., 1994). The promoter region of the *down syndrome critical region isoform 4* gene contains several binding sites for NF-AT, which are required for depolarization-dependent expression of the gene (Vihma et al., 2016). Although biochemical properties of stimulus-regulated transcription factors have not been characterized in insects, our exploratory *in silico* analysis showed structural conservation in the DNA-binding domains of stimulus-regulated transcription factors among insects and vertebrates. This suggests that insect stimulus-regulated transcription factors share DNA-binding properties similar to their vertebrate counterparts (Table 3-1 and Fig. 3-1). Structural analyses of the promoter region of *egr-B* homologs first revealed that the putative core promoter region of basal insect/crustacean *egr-B* homologs contains two conserved CREs and one SRF. Also, potential binding sites for stimulus-regulated transcription factors are enriched in the distal upstream regions of insect/crustacean *egr-B* homologs (Fig. 3*C*). Comparative structural analysis of the upstream sequences of polyneopteran *egr-B* homologs revealed two conserved CRMs, which contain binding sites for stimulus-regulated transcription factors, such as AP-1, CREB, SRF, and C/EBP. CRMs integrate the multiple transcription factors and their associated cofactors at a specific timing to perform elaborate and accurate regulation (Jeziorska et al., 2009). In fact, when we stimulated an individual intracellular signaling pathway, the expression of *Gryllus egr-B* was not induced (Fig. 2-2). Although the reason is unclear, it is possible that synergic activation of multiple signaling pathways and their downstream transcription factors is necessary to induce expression of *Gryllus egr-B* in the cricket brain. Further studies are necessary to understand the upstream signal transduction pathways involved in transcriptional activation of insect *egr-B* homologs.

In the vertebrate nervous system, *egr* family genes are involved in long-term plasticity and the formation and consolidation of long-term memory (Poirier et al., 2008). Genome-wide analysis for the direct target of Egr-1 protein in PC12 neurons revealed that a majority of Egr-1 target genes are down-regulated when *egr-1* is overexpressed, suggesting that the Egr-1 protein principally functions as a transcription suppressor (James et al., 2005). However, other studies revealed that the activity-induced Egr-1 and Egr-3 proteins up-regulate the expression of GABA_A_ receptor subunits in the hippocampal neurons to regulate homeostatic excitatory/inhibitory balance (Roberts et al., 2005; Mo et al., 2015). These controversial findings suggest that the target gene and transcriptional regulation of the Egr family proteins are determined according to cell type and/or in gene-dependent manners. In the insect nervous system, the function of the *egr* homolog, as well as its downstream target genes, is poorly understood. Further studies are needed to address the biological significance of the activity-regulated expression of the *egr* homolog in the insect brain.

## Synthesis

Reviewing Editor: Darcy Kelley, Columbia University Decisions are customarily a result of the Reviewing Editor and the peer reviewers coming together and discussing their recommendations until a consensus is reached. When revisions are invited, a fact-based synthesis statement explaining their decision and outlining what is needed to prepare a revision will be listed below. The following reviewer(s) agreed to reveal their identity: Ronald Hoy.

The reviewers concur that the paper is a valuable contribution to the literature and has potential to spearhead the application of transgenic technics into non-model (i.e. non-dipteran) insect systems in neuroethology. However, in its present form, potential impact is lessened by incomplete characterization of the transgenics; additional data are required.

Excitability data beyond just brain and suboesphageal ganglia would strengthen the manuscript. How did transformed crickets perform in behavioral tests such as courtship, grooming, and fighting? Were transformed crickets “bang sensitive” by analogy to the drosophila BS mutants, in which hyper-excitability is induced by extreme mechanical shock?

Major points: 

1. While the characterization of IEG expression in a transcriptional level by qRT-PCR is good, additional documentation such as in situ hybridization or immunostaining of egr-B expression is lacking. If egr-B expression is truly useful as a marker of neural activity, it is important to show its expression in response to neural activity by histological methods. This will be informative for researchers who use insects other than crickets, since transgenic approach has not been applied to many insect species. 

2. The authors only show one picture for each condition which raises the question of reproducibility. To show that activity-dependent GFP expression is reproducible requires quantifying GFP signal intensity or counting the number of GFP-positive cells across individuals.

3. It is not clear how well-established the transgenic reporter system, since it failed to detect behaviorally-driven neural activity (physiologically relevant neural activity) in the brain. To evaluate the reporter system, values for fold-change and statistical analyses are essential.  The main point of this paper is characterization of activity-dependent expression of known IEGs in a transcriptional level. Toning down the emphasis on the reporter system in the title, summary, and the main text woukld strengthen the paper.

Detailed comments:

1. Fig.2, Fig1-5, Fig 2-1 

In these experiments, authors tested expression of IEGs in response to PTX injection, but did not examine their expression in response to vehicle injection. It is essential to conduct qRT-PCR analyses using vehicle injected-animals as a control, to confirm PTX-induced IEG expression is neural activity-dependent. 

2. Fig 5, 6, 7 

The mRNA expression of reporter construct peaks at 90 min after stimulation. However, the authors performed GFP immunostaining at 6 or 3 h after neural activity. Time points should be justified.

If these time points are required for GFP detection, authors should show quantitative data and statistical analyses. For example, time-course analyses of immunostaining and quantification, or of western analyses. 

3. Fig 6 and 7 

Number of brains analyzed are required to quantify data and address reproducibility.

4. A flow chart of the technical procedures especially for the transgenics, within the main text, would improve the reader's ability to follow the experiments.

Minor points:

1.Typos 

P4 L38,P29 L586 

Ontogenetic -> optogenetic 

Fig1F Y axis 

hr38 -> egr 

2. Fig 6 

It seems that GFP signal intensities are variable among cells and brain regions. Can the authors discuss these points in the results? IEGs are often expressed variably among different cell-types in vertebrates. 

3. Fig1-5B C, 2-1A 

It is better to use 0 min samples, instead of naive samples, as a baseline expression, since it is difficult to know relative increase of IEG expression.
