# Supplementary material for: Immediate-Early Promoter-Driven Transgenic Reporter System for Neuroethological Research in a Hemimetabolous Insect
Source: eNeuro. 2018 Sep 4;5(4):ENEURO.0061-18.2018. doi: 10.1523/ENEURO.0061-18.2018 (PMC6140108; doi:10.1523/ENEURO.0061-18.2018)
Supplement: Table 3-1 — List of stimulus-regulated transcription factors (TFs) in mammals and their homologs in insects. Download Table 3-1, DOC file. [file sup_enu-eN-MNT-0061-18-s11.doc]

**Table 3-1. List of stimulus-regulated transcription factors (TFs) in mammals and their homologs in insects.**

| **1. TFs whose potential binding sites were searched by LASAGNA-Search 2.0** | | | | | | | |
| --- | --- | --- | --- | --- | --- | --- | --- |
| **TF families** | **Mouse homologs** | ***Drosophila* homologs** | ***Tribolium* homologs** | ***Apis* homologs** | ***Acyrthosiphon* homolog** | ***Zootermopsis* homolog** | ***Gryllus* homologs** |
| Fos | c-Fos (NP_034364.1)  FosB (NP_032062.1)  Fra-1 (NP_034365.1)  Fra-2 (NP_032063.2) | Kayak (NP_001027579.1) | NP_001164294.1 | XP_006564216.1 | XP_016663984.1 | KDR10584.1 | LC215244 |
| Jun | c-Jun (NP_034721.1)  JunB (NP_032442.1)  JunD (NP_034722.1) | Jra (NP_476586.1) | NP_001164127.1 | XP_003251036.1 | Jra-A (XP_001947556.1)  Jra-B (XP_001949916.1) | KDR21486.1 | LC215245 |
| Jun dimerization protein etc. | JDP1/BATF3 (NP_084336.1)  JDP2 (NP_001191981.1)  B-ATF (NP_058047.1)  BATF2 (XP_006531914.1) | Insect genomes do not contain homologs of these genes. | | | | | |
| Maf | c-Maf (NP_001020748.2)  MafA (NP_919331.1)  MafB (NP_034788.1)  Nrl (NP_001129546.1) | traffic jam (NP_609969.2) | XP_969910.2 | XP_006570975.1 | XP_008178362.1 | KDR08104.1 | GAIZ01020313 (*Gryllus firmus* TSA) |
|  | MafK (NP_034887.1)  MafF (NP_001291759.1)  MafG (NP_034886.1) | Maf-S (NP_611500.1) | XP_001807559.1 | XP_006563010.1 | XP_001946561.2 | KDR10857.1 | GAIZ01018353.1 (*Gryllus firmus* TSA) |
| C/EBP | C/EBPα (NP_001274443.1)  C/EBPβ (NP_001274667.1)  C/EBPδ (NP_031705.3)  C/EBPε (NP_997014.1) | slow border cells (NP_523843.1) | XP_967524.1 | XP_003251000.1 | XP_003246737.1 | KDR11649.1 | GAIZ01002687 (*Gryllus firmus* TSA) |
|  |  |  |  |  |  |  |  |
|  |  |  |  |  |  |  |  |
|  |  |  |  |  |  |  |  |
|  |  |  |  |  |  |  |  |
|  | C/EBPγ (NP_034014.1) | CG6272 (NP_648434.1) | XP_968539.1 | XP_001122278.1 | NP_001233039.1 | KDR14967.1 | GAIZ01016801 (*Gryllus firmus* TSA) |
|  | C/EBPζ (NP_001019977.1) | CG7839 (NP_648431.3) | XP_015833828.1 | XP_003250963.1 | XP_008180863.1 | not found | GAIZ01006912 and GAIZ01018921 (*Gryllus firmus* TSA) |
| CREB/ATF | OASIS (NP_036087.2) | CrebA (NP_524087.3) | CREB-A (XP_966968.2)  CREB-like (XP_973089.1) | CREB-A (XP_003250132.1)  CREB-like (XP_001121941.2) | CREB-A (XP_001948312.1)  CREB-like (XP_001949209.1) | CREB-A (KDR23733.1)  CREB-like (KDR11962.1) | CREB-like (GAIZ01013153 (*Gryllus firmus* TSA)) |
|  | CREB1 (NP_034082.1)  CREM (NP_001104320.1)  ATF1 (NP_031523.3) | CrebB (NP_001097017.1) | XP_008192794 | XP_623392.3 | XP_008186705.1 | KDR23211.1 | GAIZ01012380 and GAIZ01007852 (Gryllus firmus TSA) |
|  | ATF2 (NP_001020264.1) | dATF2 (NP_001033973.1) | XP_974257.1 | XP_003249317.1 | - | KDR15907.1 | - |
|  | ATF3 (NP_031524.2) | dATF3 (NP_620473.1) | XP_008192299.1 | XP_003251072.1 | XP_003243558.1 | KDR22659.1 | - |
|  | ATF4 (NP_001274109.1)  ATF5 (NP_109618.1) | cryptocephal (NP_524897.1) | NP_001280506.1 | XP_006562898.1 | XP_003247514.1 | KDR14457.1 | - |
|  | ATF6 (NP_001074773.1) | dATF6 (NP_995745.1) | XP_008201619.1 | XP_395889.5 | XP_008183931.1 | KDR16855.1 | GAIZ01013605 (*Gryllus firmus* TSA) |
| Egr | Egr-1 (NP_031939.1)  Egr-2 (NP_034248.2)  Egr-3 (NP_061251.1)  Egr-4 (NP_065621.1) | Stripe (NP_732289.1) | AGT37074.1 | XP_008200856.1 | XP_001943786.2 | KDR18786.1 | LC215246 |
| NF-AT | NFATc1 (NP_001157581.1)  NFATc2 (NP_035029.2)  NFATc3 (NP_035031.2)  NFATc4 (NP_001161818.1)  NFAT5 (NP_001273189.1) | dNFAT (NP_001259526.2) | - | XP_006566821.2 | - | KDR23975.1 | - |
| MEF2 | MEF2A (NP_001028885.1)  MEF2B (NP_001038949.1)  MEF2C (NP_079558.1)  MEF2D (NP_001297516.1) | dMef2 (NP_477021.1) | XP_397383.3 | XP_971771.1 | XP_003245832.1 | KDR18664.1 | GAIZ01017036 (*Gryllus firmus* TSA) |
| SRF | SRF (NP_065239.1) | blistered (NP_726438.1) | XP_016770575.1 | NP_001139383.1 | XP_008180549.2 | KDR17235.1 | - |
| **2. TFs whose potential binding sites were contained in the conserved CRMs of orthopteran *egr-B* homologs** | | | | | | | |
| **TF families** | **Mouse homologs** | ***Drosophila* homologs** | ***Tribolium* homologs** | ***Apis* homologs** | ***Acyrthosiphon* homolog** | ***Zootermopsis* homolog** | ***Gryllus* homologs** |
| AP-4 | TFAP4  (NP_112459.1) | cropped (NP_476605.1) | XP_967737.2 | XP_001122450.1 | XP_001945298.2 | KDR24214.1 | - |
| CDP/Cut | Cux1 (NP_001278162.1) | cut (NP_524764.1) | XP_015836585.1 | XP_016768219.1 | XP_003244655.1 | KDR21113.1 | - |
